# The NLRP3 inflammasome at the crossroads of innate immune signaling and cell death

**DOI:** 10.1038/s41423-026-01463-2

**Published:** 2026-09-02

**Authors:** Nanyang Xiao, Zexuan Lu, Destiny K. Cooper, Jueqi Chen

**Affiliations:** 1 Department of Microbiology, University of Chicago, Chicago, IL USA; 2 Howard Taylor Ricketts Laboratory, University of Chicago, Lemont, IL USA

**Keywords:** Inflammasome, Innate immunity, Cell death, Inflammasome, Cell death and immune response, Pattern recognition receptors

## Abstract

The NLRP3 inflammasome is a central signaling pathway of innate immunity that orchestrates host defense and inflammatory responses through the activation of proinflammatory cytokines and the induction of pyroptotic cell death. Accumulating evidence indicates that NLRP3 functions within an extensive network of innate immune and cell death pathways. Reciprocal interactions between NLRP3 and other pattern-recognition receptor pathways, including Toll-like receptors, DNA-sensing cGAS–STING signaling, and RNA-sensing RIG-I/MDA5–MAVS signaling, shape the magnitude and duration of inflammatory responses during pathogen infection. Moreover, substantial crosstalk exists between NLRP3 and other inflammasomes and programmed cell death pathways, reflecting the integrated nature of cellular stress and inflammatory signaling. In this review, we summarize recent advances in our understanding of the molecular mechanisms that mediate these interactions, focusing on shared signaling components, organelle dynamics, posttranslational modifications, and feedback regulatory circuits. We further discuss how these signaling networks contribute to infectious and inflammatory diseases and highlight key unanswered questions and emerging areas of investigation that may guide the development of therapies targeting inflammasome-associated pathologies.

## Introduction

Nucleotide-binding domain and leucine-rich repeat-containing (NLR; also known as NOD-like receptor) proteins constitute a family of intracellular innate immune sensors that regulate inflammatory responses to microbial infection and cellular stress [1, 2]. Among NLRs, NLR family pyrin domain-containing 3 (NLRP3, also known as Cryopyrin, NALP3, CIAS1, and PYPAF1) has emerged as a major focus of investigation because of its ability to detect both pathogen-associated molecular patterns (PAMPs) and damage-associated molecular patterns (DAMPs) [3]. Compared with other pattern recognition receptors (PRRs), NLRP3 can sense a remarkably diverse spectrum of stimuli, including small molecules such as the bacterial ionophore nigericin, bacterial pore-forming toxins, viral ion channels, endogenous danger signals such as extracellular ATP, and occupational particulates such as silica and microbial nucleic acids [4, 5]. This distinct feature positions the NLRP3 inflammasome as a key inflammatory signaling pathway in both host defense and inflammatory diseases, including metabolic, neurodegenerative, cardiovascular, and autoimmune diseases, making it an attractive therapeutic target [6, 7]. In addition, spontaneous gain-of-function mutations in NLRP3 in humans cause cryopyrin-associated periodic syndrome (CAPS), a spectrum of rare autosomal dominant autoinflammatory disorders characterized by constitutive inflammasome activation and excessive IL-1β production [8–10], highlighting the pathogenic importance of dysregulated inflammasome signaling.

**Fig. 1:**
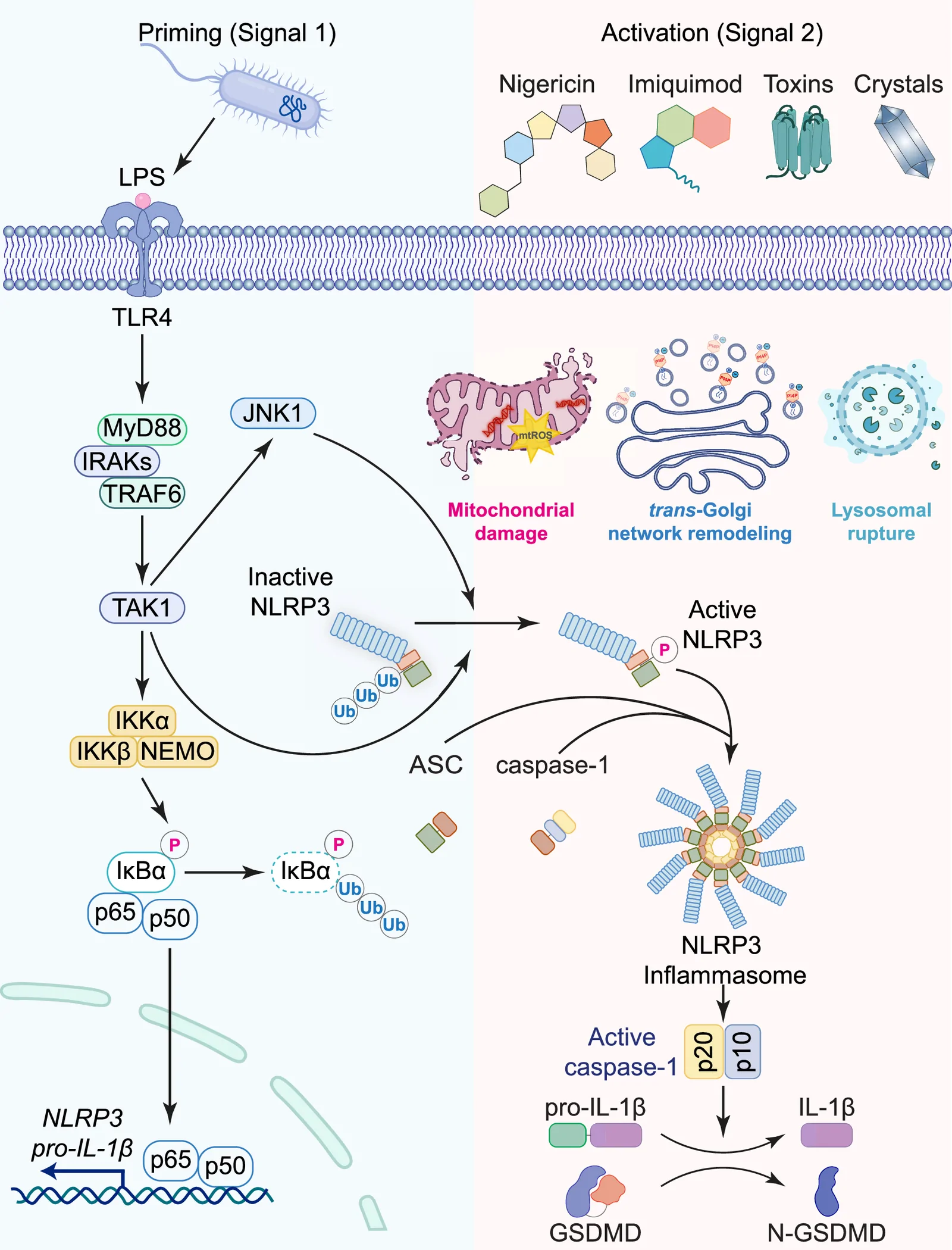
Priming and activation of the NLRP3 inflammasome. Left: Toll-like receptor signaling, such as Toll-like receptor 4 (TLR4) activation by lipopolysaccharide (LPS) from Gram-negative bacteria, provides the priming signal (signal 1) through both transcription-dependent and -independent mechanisms. For transcription-dependent mechanisms, TLR signaling leads to activation of the nuclear factor kappa B (NF-κB) signaling pathway. NF-κB, which consists of p65 and p50 subunits, induces transcriptional upregulation of NLRP3 and pro-interleukin (IL)-1β. On the other hand, transcription-independent mechanisms, which occur before transcription-dependent mechanisms, stimulate changes in post-translational modifications of NLRP3, including deubiquitination and JNK1-mediated phosphorylation, further licensing NLRP3 for activation. Right: upon activation by diverse stimuli (signal 2), NLRP3 undergoes conformational changes and oligomerization, before recruiting the adaptor protein ASC and effector caspase-1. Active caspase-1 cleaves pro-IL-1β into its mature form and processes GSDMD to generate the pore-forming N-terminal fragment, resulting in secretion of IL-1β and induction of pyroptotic cell death

NLRP3 consists of an N-terminal pyrin domain (PYD), a central NAIP, CIITA, HET-E, and TP1 (NACHT) domain, and a C-terminal leucine-rich repeat (LRR) domain. Upon stimulation by diverse agonists, NLRP3 undergoes conformational changes and oligomerization through its NACHT domain. This process exposes the PYD domain, which serves as the scaffold for inflammasome assembly [11] (Fig. 1). Activated NLRP3 recruits the adaptor protein apoptosis-associated speck-like protein containing a CARD (ASC, also known as PYCARD) through PYD–PYD interactions [12]. ASC contains both an N-terminal PYD and a C-terminal caspase recruitment domain (CARD), allowing it to function as a molecular bridge between NLRP3 and the downstream effector protease caspase-1. Upon recruitment, ASC rapidly polymerizes into large helical filaments that condense into a single supramolecular structure known as the ASC speck [13, 14]. Caspase-1 is subsequently recruited to ASCs via CARD–CARD interactions, generating catalytically active caspase-1 subunits, including p20 and p10 fragments. Activated caspase-1 cleaves the precursor forms of proinflammatory cytokines, including interleukin (IL)-1β and IL-18, into their mature forms [15]. In parallel, caspase-1 processes the pore-forming protein gasdermin D (GSDMD), liberating its N-terminal fragment, which oligomerizes within the plasma membrane to form large transmembrane pores. These pores facilitate the release of IL-1β and IL-18 and disrupt cellular ion homeostasis, ultimately leading to a proinflammatory type of cell death termed pyroptosis [16–18]. The membrane protein nerve injury-induced protein 1 (NINJ1) has been identified as a critical mediator of terminal plasma membrane rupture during pyroptosis, facilitating the release of cellular contents [19]. NLRP3 is also activated in the noncanonical inflammasome, which is initiated by direct sensing of cytosolic lipopolysaccharide (LPS) from gram-negative bacteria by caspase-4/5 in humans and caspase-11 in mice, which triggers potassium (K⁺) efflux through GSDMD pore formation, leading to NLRP3 inflammasome assembly and activation [20–22].

Given the broad nature of the stimuli capable of activating the NLRP3 inflammasome, it is not surprising that NLRP3 does not sense these stimuli through direct ligand binding. Instead, accumulating evidence has suggested that NLRP3 senses common cellular disturbances generated by diverse insults, including ion flux, organelle remodeling, and aberrantly localized molecules. Several nonmutually exclusive models have been proposed to explain inflammasome activation. K⁺ efflux, *trans*-Golgi network (TGN) remodeling, lysosomal rupture, and mitochondrial dysfunction represent the most extensively characterized mechanisms (Fig. 1). With a few exceptions, such as imiquimod (also known as R837) and CL097 [23], the majority of NLRP3 stimuli induce rapid intracellular potassium depletion, which is essential for inflammasome activation [24, 25]. Some stimuli, such as the ionophore nigericin, the membrane-spanning channel peptide gramicidin, and ATP, which acts through the purinergic receptor P2X_7_, directly trigger K⁺ efflux, whereas others, such as DNA virus infection and apoptosis induction, indirectly induce K⁺ efflux as a downstream consequence of plasma membrane permeabilization [26, 27]. Although the precise mechanisms by which K⁺ efflux licenses NLRP3 signaling remain incompletely understood, K⁺ efflux may be required either for NLRP3 recruitment to specific subcellular compartments or for subsequent ASC recruitment, depending on the nature of the activating stimulus [28, 29]. A wide range of stimuli, including both K^+^ efflux-dependent and -independent stimuli, induce the TGN to be remodeled into phosphatidylinositol-4-phosphate (PtdIns4P)-enriched vesicular structures that recruit NLRP3 through electrostatic interactions involving its polybasic region [28–31]. The recruitment of NLRP3 to the TGN is facilitated by IκB kinase (IKK)β [32, 33]. How diverse stimuli induce TGN remodeling to generate a molecular scaffold remains largely unknown. A recent study, however, provided mechanistic insights into how a specific group of bacterial toxins target and reorganize this organelle [29]. Most bacterial toxins that activate NLRP3, including hemolysin BL (HBL) [34] and nonhemolytic enterotoxin (NHE) [35] from *Bacillus cereus* and phospholipase C (PLC; also known as lecithinase) from *Clostridium perfringens* [36], remain anchored at the plasma membrane, where they drive K⁺ efflux. In contrast, cholesterol-dependent cytolysins (CDCs), the largest family of bacterial pore-forming toxins, follow a different path. Rather than remaining at the cell surface, CDCs are internalized after they form pores on the plasma membrane and translocate to the TGN [29]. They remodel organelles by peeling away PtdIns4P-negative membranes to expose PtdIns4P-positive surfaces, which then serve as docking sites for NLRP3 recruitment [29]. How other NLRP3 stimuli drive TGN remodeling to convert this organelle into an inflammasome assembly platform remains unclear. Lysosomal rupture represents another common mechanism engaged by diverse NLRP3-activating stimuli, including monosodium urate (MSU) crystals [37], amyloid-β aggregates [38], and PLC from *C. perfringens* [36]. Lysosomal destabilization leads to the release of lysosomal contents, such as proteolytic enzymes (cathepsins), into the cytosol, promoting NLRP3 activation through mechanisms that remain incompletely understood [37, 39–41]. Finally, multiple models have been proposed for the role of mitochondria in NLRP3 signaling. Initial studies suggested that the recruitment of NLRP3 to mitochondria [42], the production of mitochondrial reactive oxygen species (ROS) [23, 42], and mitochondrial damage [43–45] are critical events leading to NLRP3 activation, although conflicting results have been reported for some of these proposed mechanisms [46–48]. Subsequent studies demonstrated that ROS-induced mitochondrial DNA promotes NLRP3 inflammasome assembly [43, 49]. More recently, alterations in mitochondrial ATP production have been identified as important signals that act in parallel with other key cellular events, including K^+^ efflux and TGN remodeling [50]. In addition to those discussed above, several other mechanisms, including calcium (Ca^2+^) signaling [25, 51–53], disruption of endoplasmic reticulum–endosome membrane contact sites (EECS) [54, 55], and endoplasmic reticulum (ER) stress [56], have been implicated in NLRP3 activation. The breadth of proposed mechanisms for NLRP3 activation reflects the remarkable diversity of stimuli that trigger this inflammasome.

Increasing evidence suggests that the NLRP3 inflammasome is extensively integrated with other innate immune and cell death signaling networks, enabling cells to integrate diverse microbial, metabolic, and stress-derived signals into coordinated inflammatory responses. Understanding these interactions is therefore essential for deciphering the context-dependent functions of NLRP3 in immunity and disease. In this review, we discuss the molecular mechanisms underlying the interplay between NLRP3 signaling and Toll-like receptor (TLR) signaling, nucleic acid-sensing pathways, other inflammasomes, and programmed cell death pathways, with an emphasis on the mechanisms that coordinate inflammatory responses under physiological and pathological conditions.

### The Nlrp3 inflammasome and other innate immune sensing pathways

#### Toll-like receptor signaling in NLRP3 priming

Toll-like receptors (TLRs) are a family of germline-encoded PRRs that serve as a critical first line of defense against invading pathogens. In mammals, thirteen TLRs have been identified, with TLR1–TLR10 expressed in humans and TLR1–TLR9 together with TLR11–TLR13 expressed in mice [57]. TLRs are type I transmembrane proteins composed of an extracellular N-terminal LRR domain responsible for ligand recognition, a transmembrane domain, and a conserved intracellular Toll/interleukin-1 receptor (TIR) domain that mediates downstream signaling [58].

TLRs are strategically localized either on the plasma membrane or within endosomal compartments, enabling the recognition of extracellular and intracellular microbial products. Plasma membrane TLRs primarily recognize microbial membrane components. TLR4 detects LPS [59–62] through a receptor complex containing MD-2 and CD14 [63–66]. TLR2 forms heterodimers with either TLR1 or TLR6 to recognize a broad range of bacterial and fungal lipoproteins [67–69]. Specifically, the TLR1–TLR2 heterodimer senses triacylated lipoproteins [70], whereas the TLR2–TLR6 heterodimer recognizes diacylated lipoproteins [71]. TLR5 recognizes bacterial flagellin, the major structural protein of flagella [72, 73]. In contrast, endosomal TLRs are transported to endosomes facilitated by the chaperone protein UNC93B1 [74] or alternative trafficking mechanisms [75] and specialize in sensing microbial nucleic acids. The double-stranded RNA (dsRNA), a common viral replication intermediate, is detected in TLR3 [76]. TLR7 and TLR8 recognize single-stranded RNA (ssRNA) enriched in viral genomes [77–79], whereas TLR9 senses CpG DNA motifs frequently found in bacterial DNA [80]. In mice, TLR13 recognizes a conserved sequence within bacterial 23S ribosomal RNA [81, 82].

Ligand engagement induces conformational changes and the dimerization of TLR receptors, increasing the proximity of the intracellular TIR domains [83]. This event initiates the assembly of signaling complexes through interactions with adaptor proteins. Four major adaptors participate in TLR signaling: myeloid differentiation primary response protein 88 (MyD88), TIR-domain-containing adaptor protein (TIRAP, also known as MAL), TIR-domain-containing adaptor-inducing interferon-β (TRIF), and TRIF-related adaptor molecule (TRAM) [84–91]. Upon receptor activation, MyD88 oligomerizes with members of the interleukin-1 receptor-associated kinase (IRAK) family [92, 93], leading to the recruitment of the E3 ubiquitin ligase tumor necrosis factor receptor-associated factor 6 (TRAF6) [94]. TRAF6 mediates the ubiquitin-dependent recruitment and activation of transforming growth factor β-activated kinase 1 (TAK1). Activated TAK1 subsequently stimulates two major downstream pathways. First, TAK1 phosphorylates the IKK complex, which consists of IKKα, IKKβ, and NEMO (IKKγ), leading to the phosphorylation and degradation of the IκB protein [95, 96]. This process results in the release of NF-κB transcription factors such as p65 and p50, allowing their translocation into the nucleus and the induction of the expression of proinflammatory genes such as tumor necrosis factor (TNF), IL-6, IL-1β, and numerous chemokines [58]. Second, TAK1 activates mitogen-activated protein kinase (MAPK) cascades, including the extracellular signal-regulated kinase (ERK), c-Jun N-terminal kinase (JNK), and p38 pathways, which stimulate AP-1 family transcription factors and further increase inflammatory gene expression [96, 97]. These signaling networks not only provide immediate protection against pathogens but also orchestrate the development of effective adaptive immune responses.

NLRP3 inflammasome activation is a two-step process involving a priming signal (signal 1) and an activation signal (signal 2) [3]. Prior to the activation step triggered by the diverse stimuli discussed above, a priming signal is required to activate NF-κB signaling to license NLRP3 for activation. This priming signal is typically provided by TLR signaling (Fig. 1). An early study revealed that stimulation of multiple TLRs, including TLR2, TLR3, TLR4, and TLR7, did not directly activate the NLRP3 inflammasome but instead primed macrophages for subsequent NLRP3 activation in response to a second stimulus [98]. In contrast to most other inflammasome sensors, which are constitutively expressed in cells capable of supporting their activation, NLRP3 is usually expressed at relatively low levels under basal conditions [98]. TLR-induced NF-κB activation promoted NLRP3 transcription, thereby increasing NLRP3 protein abundance and promoting subsequent inflammasome activation by diverse stimuli, including ATP, pore-forming toxins, crystals, and particulate matter [99]. Consistently, overexpression of NLRP3 in either *Nlrp3*^−/−^ bone marrow-derived macrophages (BMDMs) [98, 100] or reconstituted nonmyeloid cell lines [28, 29] bypassed the requirement for a priming signal. In addition, NF-κB activation induced the transcription of pro-IL-1β, the inactive precursor of IL-1β and an essential substrate for inflammasome-mediated cytokine processing [99]. In addition to TLR signaling, TNF-α also served as a priming signal through activation of the TNF–NF-κB pathway [101]. Because many microbial pathogens can provide both TLR ligands and NLRP3-activating stimuli, a single pathogen can often supply both the priming and activation signals required for NLRP3 inflammasome activation under physiological conditions.

In addition to promoting NF-κB-dependent transcription, several nontranscriptional mechanisms through which TLR signaling facilitates NLRP3 inflammasome activation have been proposed. Acute TLR stimulation activated IRAK1, which was required for the immediate responsiveness of NLRP3 to ATP before significant NLRP3 protein induction occurred [98, 102]. Similarly, acute LPS stimulation enabled NLRP3 activation within minutes before substantial induction of NLRP3 or pro-IL-1β expression occurred [103]. These findings indicated the existence of a nontranscriptional priming pathway that modified preexisting NLRP3 to render it activation competent. One study revealed that NLRP3 was constitutively inhibited by ubiquitination, whereas TLR signaling promoted its deubiquitination, thereby alleviating this inhibition [100]. Another study revealed that TLR stimulation rapidly activated JNK1, which phosphorylated NLRP3 at Ser194 in mice (Ser198 in humans) [104]. This phosphorylation occurred independently of NLRP3 protein induction and promoted the self-association of NLRP3, facilitating its transition from an inactive state to an active state [104]. Finally, in some cell types, such as human monocytes, TLR-mediated priming was sufficient to activate the NLRP3 inflammasome because of constitutive autocrine ATP release, which served as the activation signal [105, 106].

#### Crosstalk between cGAS–STING signaling and the NLRP3 inflammasome

The cyclic GMP–AMP synthase (cGAS)–stimulator of interferon genes (STING) pathway is a central mechanism through which mammalian cells detect cytosolic double-stranded DNA (dsDNA). Because DNA is normally confined to the nucleus and mitochondria, its presence in the cytoplasm serves as a potent indicator of viral infection, bacterial invasion, mitochondrial damage, genomic instability, and cellular stress [107]. The core sensor of this pathway, cGAS, is a cytosolic nucleotidyltransferase that binds dsDNA directly through electrostatic interactions with the sugar-phosphate backbone in a sequence-independent manner [108]. DNA binding induces conformational changes in cGAS that promote dimerization and assembly into higher-order oligomeric complexes on DNA substrates [109, 110]. This DNA-driven activation enables cGAS to catalyze the synthesis of the cyclic dinucleotides 2′3′-cyclic GMP–AMP (2′3′-cGAMP) from ATP and GTP [111–115].

cGAMP is sensed by STING (also known as MITA, MPYS and ERIS), an endoplasmic reticulum (ER)-resident transmembrane adaptor protein [116–118]. Binding of cGAMP induces major conformational rearrangements in STING that promote its oligomerization and activation [119]. Activated STING exits the ER and is translocated to the ER-Golgi intermediate compartment (ERGIC) and Golgi apparatus [120–122]. At the Golgi, STING serves as a scaffold for the recruitment and activation of TANK-binding kinase 1 (TBK1) [123]. Activated TBK1 phosphorylates both STING and the transcription factor interferon regulatory factor 3 (IRF3), thereby driving the induction of type I interferons and interferon-stimulated genes [124, 125]. In parallel, STING activation promotes the recruitment of the IKK complex, leading to NF-κB activation and the subsequent expression of proinflammatory cytokines and chemokines [120].

**Fig. 2:**
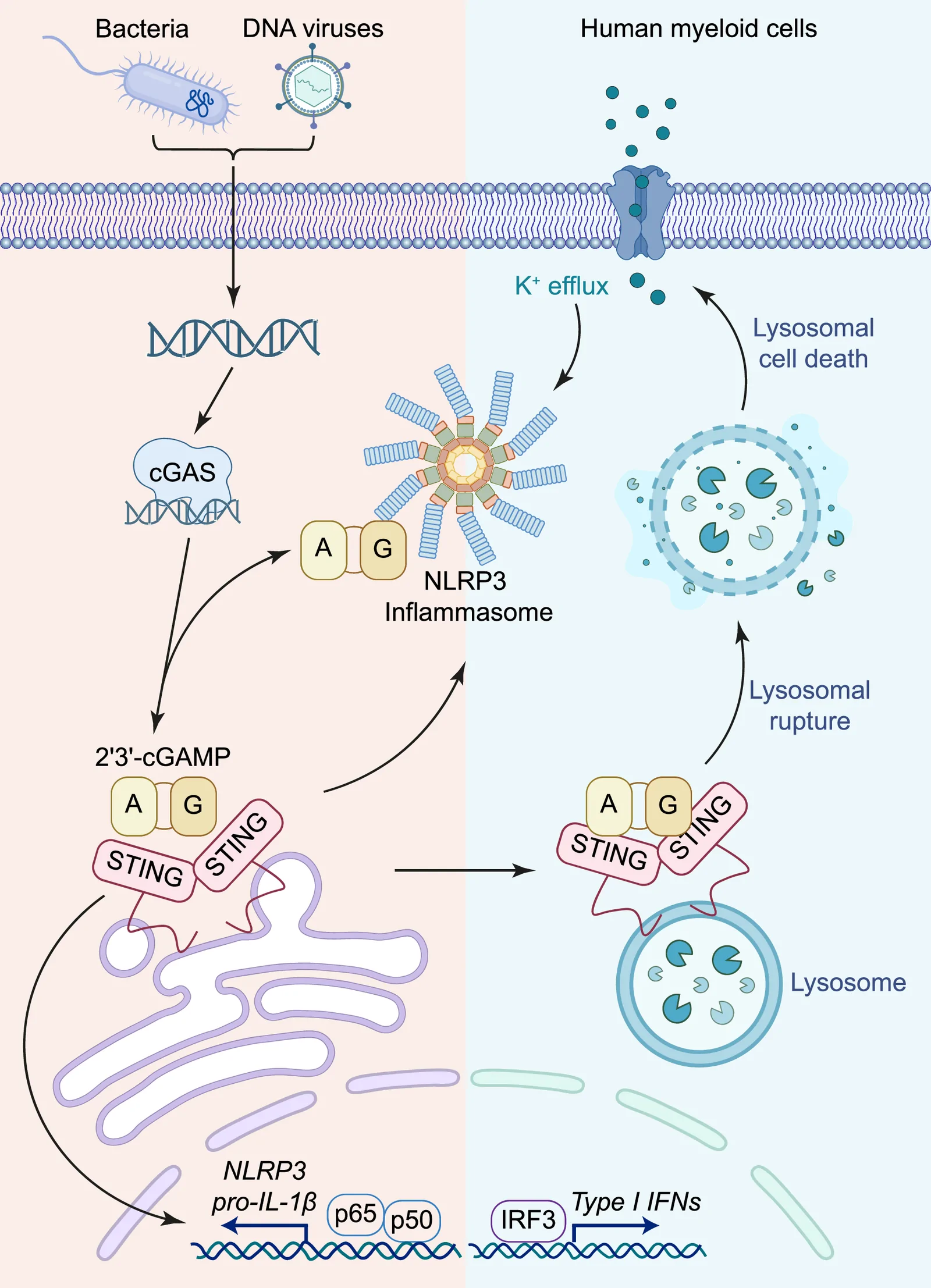
Crosstalk between cGAS–STING signaling and the NLRP3 inflammasome. Left: cytosolic DNA derived from bacteria or DNA viruses activates cGAS, which generates 2′3′-cyclic GMP–AMP (2′3′-cGAMP). In addition to functioning as a second messenger that activates STING, 2′3′-cGAMP also promotes assembly of the NLRP3 inflammasome in both STING-dependent and -independent manners. Right: in human myeloid cells, activation of cGAS–STING signaling triggers trafficking of STING to lysosomes, leading to lysosomal rupture. Subsequent lysosomal cell death results in potassium (K⁺) efflux, which promotes NLRP3 inflammasome activation

In addition to serving as a second messenger in the cGAS–STING pathway, cGAMP was found to act as a mediator of inflammasome priming and activation [126] (Fig. 2, left). Cytosolic delivery of 2′3′-cGAMP induced the expression of NLRP3 and pro-IL-1β through STING-dependent type I interferon signaling. In addition, it promoted caspase-1 activation and IL-1β/IL-18 secretion in LPS-primed macrophages and dendritic cells. These effects were dependent on both NLRP3 and the DNA inflammasome sensor absent in melanoma 2 (AIM2, also known as PYHIN4). Consistently, intracellular cGAMP colocalized with NLRP3, AIM2, and other inflammasome components [126], suggesting that cGAMP may facilitate inflammasome assembly. Although the underlying mechanism remains unclear, cGAMP-induced activation of NLRP3 and AIM2 inflammasomes was only partially dependent on STING [126], indicating that cGAMP may also be sensed by an as yet unidentified protein.

A recent study revealed that cytosolic DNA activated the NLRP3 inflammasome in human myeloid cells via the cGAS–STING pathway (Fig. 2, right) (Table 1), in contrast to murine myeloid cells, in which cytosolic DNA activated the AIM2 inflammasome [26]. In human myeloid cells, infection with the gram-negative bacterium *Francisella novicida* or the DNA virus vaccinia virus activated cGAS–STING signaling and triggered the trafficking of STING to lysosomes, resulting in lysosomal membrane permeabilization and lysosomal cell death. This process initiated K^+^ efflux, which served as the proximal trigger for NLRP3 activation [26]. Synthetic cGAMP alone was sufficient to induce NLRP3-dependent IL-1β secretion, confirming that STING activation is a critical upstream event for inflammasome activation. Another study revealed that the cGAS–STING pathway promoted NLRP3 inflammasome activation through a distinct mechanism in THP-1 cells, a human monocyte/macrophage-like cell line [127] (Table 1). Upon infection with the DNA virus HSV-1, activated STING interacted with the NACHT and LRR domains of NLRP3. This interaction reduced NLRP3 polyubiquitination and promoted its recruitment to the ER, leading to subsequent inflammasome activation [127].

**Table 1:** Cell type-specific crosstalk between the NLRP3 inflammasome and other signaling pathways

Cell Type	Components	Signaling	Outcome	References
Human myeloid cells	cGAS–STING	cGAS–STING activation → lysosomal cell death → K⁺ efflux	NLRP3 activation	[26]
THP-1 (human monocyte/macrophage-like cell line)	cGAS–STING	cGAS–STING activation → STING interaction with NLRP3 → reduced NLRP3 polyubiquitination → NLRP3 recruitment to the ER	NLRP3 activation	[127]
WM35 and WM115 (melanoma cell lines)	Human NLRP1	Human NLRP1 negatively regulated NF-κB activity	Reduced NLRP3 priming	[211]
THP-1 (human monocyte/macrophage-like cell line) 1205Lu and HS294T (metastatic human melanoma cell lines)	Human NLRP1	Human NLRP1 positively regulated NF-κB activity	Enhanced NLRP3 priming	[211]
Bone-marrow-derived macrophages (BMDMs)	Caspase-8 and FADD	Caspase-8 and FADD facilitated TLR-mediated priming	Enhanced NLRP3 priming	[48, 331]
Dendritic cells	Caspase-8	Caspase-8 deficiency enabled LPS priming alone to trigger NLRP3 activation	NLRP3 activation by LPS alone when caspase-8 is absent	[350]
Primary tubular epithelial cells (TECs)	Caspase-8 and caspase-3	TNFα/cycloheximide → extrinsic apoptosis → NLRP3- and ASC-mediated caspase-8 and caspase-3 cleavage	Apoptosis induction promoted by NLRP3 activation	[364]

Emerging evidence also suggests that NLRP3 inflammasome signaling regulates the cGAS–STING pathway. cGAS was identified as a direct substrate of caspase-1 during DNA virus infection in immortalized bone marrow-derived macrophages (iBMDMs) treated with LPS and ATP [128]. This cleavage disrupted the catalytic activity of cGAS and suppressed downstream type I interferon production [128]. Because type I interferon can inhibit inflammasome activity [129], this NLRP3-dependent downregulation of cGAS–STING signaling may represent a host strategy to amplify inflammasome signaling in response to pathogen invasion. Similarly, the NLRP3 inflammasome elicited by the positive-sense ssRNA virus Zika virus also suppressed cGAS activity [130]. In this context, it was proposed that Zika virus might exploit NLRP3 activation to antagonize cGAS–STING-mediated antiviral type I interferon responses [130]. Another study, however, proposed an alternative model for inflammasome-mediated regulation of cGAS–STING signaling [131]. In this study, caspase-1 activation induced by either NLRP3 stimuli, including nigericin and ATP, or AIM2 stimuli, including poly(dA:dT) and *F. novicida*, failed to reduce cGAS protein levels in BMDMs [131]. Instead, K⁺ efflux triggered by either GSDMD-mediated pore formation or NLRP3 stimulation alone was sufficient to suppress cGAS activity and type I interferon responses [131]. Finally, in contrast to the prevailing view that inflammasome activation suppresses cGAS–STING signaling, NLRP3 activation in colorectal cancer cells enhanced cGAS–STING signaling and promoted CD8⁺ T-cell-mediated antitumor immunity [132], highlighting context-dependent crosstalk between these pathways.

#### Crosstalk between RIG-I/MDA5–MAVS signaling and the NLRP3 inflammasome

Retinoic acid-inducible gene I (RIG-I)-like receptors (RLRs) constitute a major cytosolic surveillance system for the detection of viral RNA. The RLR family consists of three members: RIG-I (also known as DDX58), melanoma differentiation-associated gene 5 (MDA5, also known as IFIH1), and laboratory of genetics and physiology 2 (LGP2, also known as DHX58) [133]. Among these receptors, RIG-I and MDA5 function as the principal RNA sensors, whereas LGP2 serves primarily as a regulator of RLR signaling [134–139]. RIG-I and MDA5 share a common domain architecture consisting of two CARDs, a central helicase domain, and a C-terminal RNA-binding domain. These receptors recognize distinct classes of viral RNA. RIG-I preferentially detects short dsRNA molecules bearing 5′-triphosphate or 5′-diphosphate termini, molecular signatures that are frequently generated during the replication of many negative-strand and positive-strand RNA viruses [140, 141]. In contrast, MDA5 preferentially recognizes long dsRNA structures produced during infection by picornaviruses and other RNA viruses [142, 143]. Binding to viral RNA induces conformational rearrangements that expose CARDs and promote receptor oligomerization [144].

Activated RIG-I and MDA5 subsequently engage the mitochondrial antiviral signaling protein MAVS (also known as IPS-1, VISA, and Cardif) through CARD–CARD interactions [145–148]. MAVS is anchored mainly on the outer mitochondrial membrane but has also been reported to localize to peroxisomes and mitochondria-associated membranes [149]. Upon activation, MAVS undergoes higher-order oligomerization into filamentous signaling assemblies with prion-like properties [150, 151]. These MAVS polymers recruit multiple adaptor proteins and signaling enzymes, creating a platform for signal amplification. Downstream of MAVS, members of the tumor necrosis factor receptor-associated factor (TRAF) family, including TRAF2, TRAF3, TRAF5, and TRAF6, assemble ubiquitin-dependent signaling complexes that activate distinct transcriptional pathways [152–155]. The kinases TBK1 and IKKε are recruited and phosphorylate the interferon regulatory factors IRF3 and IRF7 [156, 157]. Phosphorylated IRF3 and IRF7 dimerize and translocate to the nucleus, where they drive the expression of type I interferons. Concurrently, the MAVS-dependent recruitment of TRAF6 and related signaling components activates the TAK1 kinase complex and the canonical IKK complex, leading to the activation of the transcription factor NF-κB and the induction of inflammatory cytokines [158].

In addition to its role in the antiviral RNA-sensing pathway, MAVS has been reported to function as a crucial regulator of the NLRP3 inflammasome, although its precise contribution remains debated [47]. An early study reported that MAVS interacted with NLRP3 and mediated its tethering to mitochondria, thereby promoting inflammasome activation [159]. *Mavs*^−/−^ BMDMs exhibited reduced caspase-1 and IL-1β processing following stimulation with nigericin or ATP [159]. However, several subsequent studies failed to report defects in NLRP3 inflammasome activation in *Mavs*^−/−^ BMDMs upon stimulation with nigericin, ATP, MSU, or silica [48, 160, 161]. Instead, activation of MAVS through RIG-I/MDA5 signaling, either by poly(I:C) transfection or infection with the negative-sense ssRNA virus Sendai virus, was found to facilitate NLRP3 inflammasome activation in BMDMs and THP-1 cells [160, 161]. Although the underlying mechanisms remain incompletely resolved, K^+^ efflux triggered by MAVS-induced plasma membrane permeabilization has been shown to be critical [160]. These findings suggest that MAVS is not a core component required for NLRP3 inflammasome activation in general but can function as an upstream signal that links RNA-sensing pathways to NLRP3 activation.

### The Nlrp3 inflammasome and other inflammasome pathways

#### Crosstalk between the NAIP–NLRC4 and the NLRP3 inflammasome

The NLR family of apoptosis inhibitory proteins (NAIP, also known as BIRC1 and NLRB1)–NLR family CARD domain-containing protein 4 (NLRC4, also known as Ipaf, CARD12, and CLAN1) inflammasome is a receptor–adaptor inflammasome that detects bacterial virulence factors delivered into the host cell cytosol [162]. Human and mouse NAIPs differ markedly in gene number and ligand specificity. Mice encode multiple NAIP paralogs, including *Naip1*, *Naip2*, *Naip5*, *Naip6*, and others. In contrast, humans possess a single *NAIP* gene [163, 164]. NAIP proteins contain three N-terminal baculovirus inhibitor of apoptosis repeat (BIR) domains, a NACHT domain, and a C-terminal LRR domain, whereas NLRC4 consists of an N-terminal CARD, a NACHT domain, and a C-terminal LRR domain [163, 165]. In this pathway, NAIPs function as sensor proteins that detect PAMPs and subsequently recruit NLRC4 to initiate inflammasome assembly and signaling. In mice, NAIP1 senses the needle protein of the bacterial type III secretion system (T3SS), NAIP2 recognizes the inner rod protein of the T3SS, and NAIP5/6 detects bacterial flagellin [166–171]. The ligand specificity for human NAIP remains incompletely defined. Early studies suggested that human NAIP recognizes only the T3SS needle protein [172, 173] or flagellin [174]. However, a later study revealed that human NAIP is capable of sensing all three classes of bacterial ligands [175].

The structural basis of NAIP–NLRC4 inflammasome assembly has been extensively characterized [165, 176–181]. Upon ligand binding, NAIP undergoes a major conformational change that exposes its oligomerization interfaces and nucleates the assembly of NLRC4 protomers. Activated NLRC4 subsequently recruits additional NLRC4 molecules, resulting in the formation of a wheel-shaped inflammasome complex. The CARD of NLRC4 directly recruits caspase-1, although ASC can also be incorporated to enhance cytokine maturation [182, 183]. While several studies have reported that Ser533 phosphorylation is critical for NLRC4 activation [184, 185], a subsequent study reached different conclusions [186], leaving its functional importance unresolved.

**Fig. 3:**
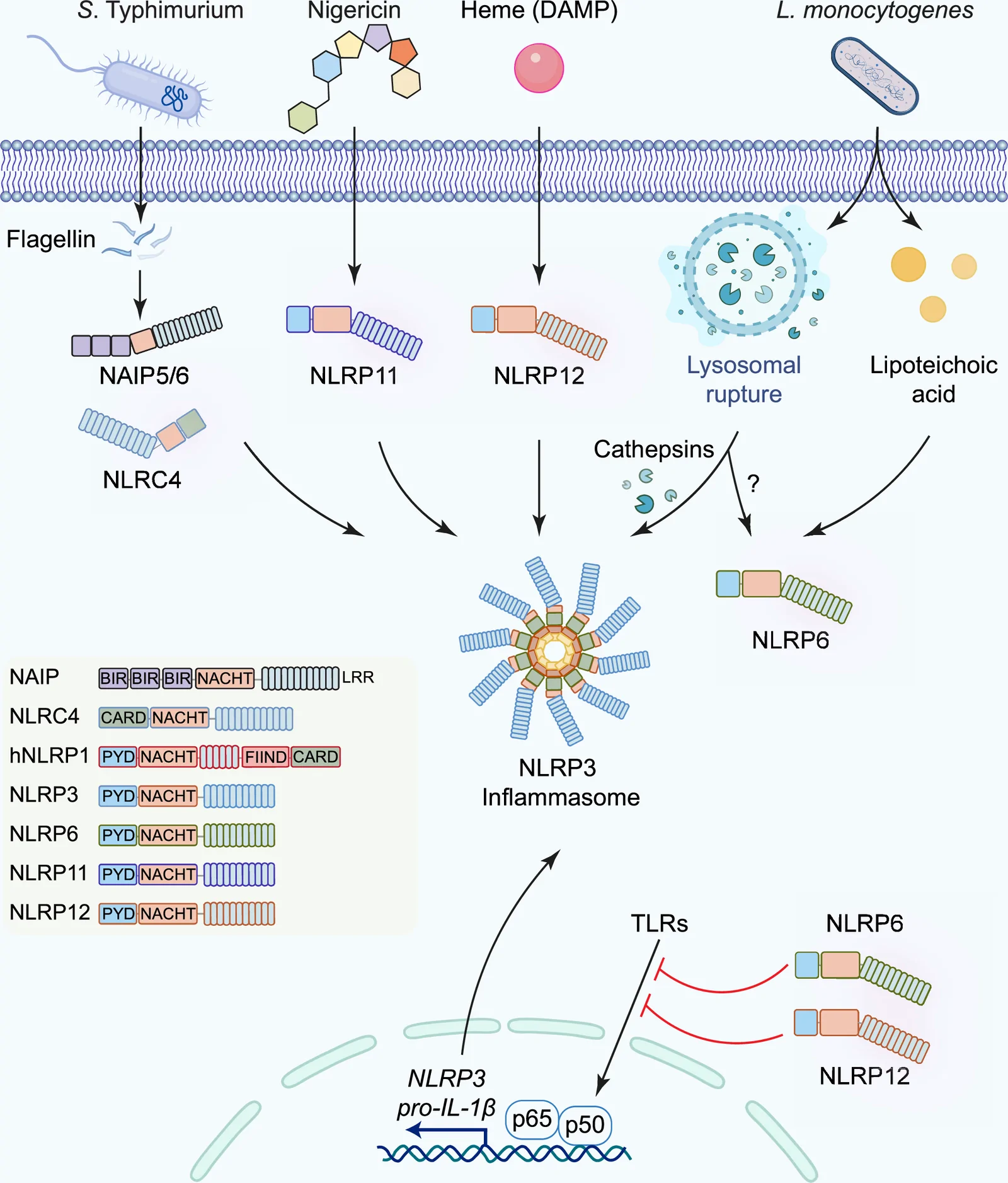
Crosstalk between the NLRP3 inflammasome and other inflammasome pathways. Multiple inflammasome pathways, including NAIP5/6–NLRC4 sensing of bacteria-derived flagellin, NLRP11 activation downstream of nigericin treatment, and NLRP12 activation in response to heme, promote the assembly and activation of the NLRP3 inflammasome. In addition, both NLRP6 and NLRP12 negatively regulate NF-κB signaling and thus suppress NLRP3 inflammasome priming. *Listeria monocytogenes* infection triggers NLRP6 inflammasome activation through lipoteichoic acid or lysosomal rupture. Lysosomal rupture also mediates NLRP3 activation through the release of cathepsins, whereas the lysosomal factor(s) responsible for NLRP6 activation remain unclear. The inset illustrates the domain architecture of representative inflammasome sensors. DAMP, damage-associated molecular pattern. hNLRP1, human NLRP1. Domain abbreviations are as follows: BIR baculoviral inhibitor of apoptosis protein repeat, NACHT NAIP, CIITA, HET-E, and TP1 domain, LRR leucine-rich repeat, CARD caspase recruitment domain, PYD pyrin domain

The NAIP–NLRC4 inflammasome can promote NLRP3 activation after bacterial infection (Fig. 3). Following infection with the gram-negative bacterium *Salmonella enterica* serovar Typhimurium (*S*. Typhimurium), NAIP–NLRC4 and NLRP3 synergistically coordinated host antimicrobial immunity. *S*. Typhimurium-induced caspase-1 cleavage and IL-1β secretion were only partially reduced in *Nlrp3*^−/−^ BMDMs and *Nlrc4*^−/−^ BMDMs but were completely abolished in *Nlrp3*^−/−^*Nlrc4*^−/−^ BMDMs, indicating that NLRP3 and NAIP–NLRC4 inflammasomes play synergistic roles in supporting inflammasome activation during *S*. Typhimurium infection [187]. Consistent with these findings, superresolution microscopy revealed that NLRP3 and NLRC4 each formed distinct ring-like structures of smaller diameter that were enclosed within the same larger ASC ring in *S*. Typhimurium-infected THP-1 cells, suggesting that both proteins can be incorporated into a single inflammasome complex [182]. Consistent with earlier reports showing that NLRC4 deficiency largely abolished *S*. Typhimurium-induced inflammasome activation [188–190], NLRC4 mediated the early phase of inflammasome activation (2 hours post-infection), whereas NLRP3 contributed only at later time points (24 h post-infection) [182]. Whether this delayed activation of NLRP3 reflects differences in ligand availability or the kinetics of inflammasome assembly remains unclear. Consistent with the in vitro findings, both NLRP3 and NAIP–NLRC4 contributed to host defense against *S*. Typhimurium in a sublethal murine salmonellosis model [182]. Mechanistically, NLRC4 was reported to recruit NLRP3 to the inflammasome complex through its NACHT domain [185]. In contrast, NAIP–NLRC4 inflammasome activation induced by flagellin did not require NLRP3 either in vitro or in vivo [186]. These findings suggest that NLRC4 can facilitate NLRP3 inflammasome assembly under specific infectious conditions, whereas NLRP3 is dispensable for canonical NLRC4 inflammasome activation.

#### Crosstalk between NLRP1 and the NLRP3 inflammasome

NLRP1 (also known as NALP1 and CARD7) was among the first inflammasome sensors identified [191]. Like NAIP, NLRP1 exhibits substantial species-specific differences. While humans possess a single *NLRP1* gene located on chromosome 17 [192], mice contain multiple paralogous *Nlrp1* genes, including *Nlrp1a*, *Nlrp1b*, and *Nlrp1c*, although the number and functionality of these genes vary among mouse strains [193, 194]. NLRP1B is the best-characterized murine NLRP1 inflammasome sensor, whereas NLRP1C is generally considered nonfunctional in many laboratory strains [195]. Structurally, human NLRP1 contains an N-terminal PYD followed by a NACHT domain, LRRs, a function-to-find domain (FIIND), and a C-terminal CARD. In contrast, NLRP1B lacks an N-terminal PYD. Both human and murine NLRP1 proteins undergo constitutive autoproteolysis within the FIIND, generating an N-terminal fragment and a C-terminal fragment containing the UPA subdomain of FIIND and the CARD [196]. The C-terminal fragment remains noncovalently associated with the N-terminal fragment in an autoinhibited state. Diverse stimuli activate NLRP1 by inducing proteasomal degradation of the N-terminal portion, thereby liberating the bioactive UPA–CARD fragment that nucleates ASC and caspase-1 assembly [197, 198].

Human NLRP1 is activated by enteroviral proteases, particularly picornavirus 3C proteases, which cleave the N-terminus of human NLRP1 and trigger degradation-mediated activation [199, 200]. Human NLRP1 also responded to UVB-induced ribotoxic stress through the ZAKα kinase pathway [201], the DNA virus Kaposi’s sarcoma-associated herpesvirus (KSHV) protein open reading frame (ORF)45 [202], and virus-derived dsRNA [203]. In contrast, murine NLRP1B functions as a sensor of pathogen enzymes such as anthrax lethal factor (LF), a zinc-dependent metalloprotease produced by the gram-positive bacterium *Bacillus anthracis* [204, 205]. LF cleaved NLRP1B and initiated N-terminal degradation, thus alerting host cells to bacterial invasion. Additional regulation occurs through dipeptidyl peptidase 9 (DPP9), an intracellular serine protease that functions as a major negative regulator of the NLRP1 inflammasome [206, 207]. DPP9 binds the FIIND region and sequesters the active C-terminal fragment, thereby acting as a checkpoint against spontaneous activation [208, 209]. Consistent with the importance of this negative regulation, several germline NLRP1 mutations have been identified in patients with autoinflammatory and skin disorders, leading to aberrant inflammasome activation [210].

Human NLRP1 affects NLRP3 signaling at both the priming and activation stages. In addition to driving pyroptosis and proinflammatory cytokine production through inflammasome assembly, NLRP1 also exerts context-dependent effects on NF-κB signaling [211], suggesting the potential regulation of the priming step of NLRP3 inflammasome activation. Human NLRP1 knockdown promoted NF-κB activity in the human melanoma cell lines WM35 and WM115 but decreased NF-κB activity in THP-1 cells and the metastatic human melanoma cell lines 1205Lu and HS294T [211] (Table 1). Mechanistically, NLRP1 inflammasome activation led to caspase-1-dependent processing and release of mature IL-1β, which in turn engaged IL-1 receptors to regulate NF-κB signaling [211]; however, the mechanism through which NLRP1 inflammasome activation exerts opposite effects in different cell types remains unknown. In addition, NLRP1 was reported to sense nigericin, a well-characterized NLRP3 stimulus, in human keratinocytes [212, 213]. By depleting intracellular K^+^, nigericin activated the ribotoxic stress response sensor kinase ZAKα, leading to the activation of p38 and JNK signaling, which were required for human NLRP1 activation [201]. Since a variety of other NLRP3 stimuli also induce K^+^ efflux [24], whether NLRP1 responds to these stimuli and how it synergizes with the NLRP3 inflammasome during pathogen infection remain to be determined.

#### Crosstalk between NLRP6 and the NLRP3 inflammasome

NLRP6 (also known as NALP6, PYPAF5, and PAN3) is predominantly expressed in intestinal epithelial cells [214, 215], where it contributes to mucosal homeostasis, antimicrobial defense, and host–microbiota interactions [216, 217]. The role of NLRP6 in microbiota recognition and intestinal homeostasis has been extensively investigated [218–222], although conflicting findings have been reported [223, 224]. In addition to the microbiota, a variety of pathogen-derived stimuli have been proposed to activate NLRP6. dsRNAs associated with enteric RNA viruses activated both human and mouse NLRP6 inflammasomes through liquid–liquid phase separation [225–227]. Infection with gram-positive bacteria, such as *Listeria monocytogenes*, induced potent caspase-11 activation and cytokine release through the NLRP6 inflammasome in BMDMs [228]. In contrast, an *L. monocytogenes* strain lacking lipoteichoic acid (LTA) synthase failed to induce caspase-11 activation [228]. Together with the observation that NLRP6 interacted with LTA through its LRR domain, these findings suggest that LTA serves as a direct ligand for NLRP6 in macrophages [228]. A recent study [214], however, proposed an alternative mechanism of NLRP6 activation during *L. monocytogenes* infection. This study revealed that NLRP6 was highly expressed in intestinal epithelial cells but not in BMDMs and that transfection with LTA alone failed to activate the inflammasome. Instead, NLRP6 activation in intestinal epithelial cells was triggered by endolysosomal rupture following bacterial escape into the cytosol. Sterile endolysosomal damage caused by the lysosomotropic agent L-leucyl-L-leucine methyl ester (LLOMe) also activated the NLRP6 inflammasome [214]. Interestingly, endolysosomal rupture is also a common mechanism underlying NLRP3 inflammasome activation by particulate stimuli such as MSU crystals [37], amyloid-β [38], and the antimicrobial peptide LL-37 [229]. Whether NLRP6 and NLRP3 employ similar or distinct mechanisms to sense endolysosomal damage remains unknown (Fig. 3). For example, the release of the lysosomal cysteine protease cathepsin has been proposed to be essential for NLRP3 activation following lysosomal rupture [37, 40, 41], whereas its role in NLRP6 activation has yet to be determined.

NLRP6 also exerts inflammasome-independent effects on NF-κB and MAPK signaling [230, 231], complicating the interpretation of in vivo functions. During *L. monocytogenes* infection, NLRP6 deficiency in macrophages and neutrophils led to elevated levels of phosphorylated IκB and ERK1/2 [231]. Similarly, LPS priming upregulated NLRP6 expression in human periodontal ligament cells, and NLRP6 knockdown enhanced NF-κB and ERK activation [232], suggesting that TLR signaling induces the activity of NLRP6 as a negative feedback regulator to restrain excessive inflammatory signaling, which is often associated with cancers, autoimmune diseases, and other health problems. Consequently, NLRP6 depletion increased NLRP3 expression in LPS-primed cells [232], indicating that NLRP6 negatively regulates the priming stage of NLRP3 inflammasome activation. Consistently, increased hepatic NLRP3 expression was observed in *Nlrp6*^−/−^ mice [233]. Further evidence from alkali-burn models supported a regulatory axis in which NLRP6 suppressed NF-κB signaling and consequently limited NLRP3 expression [234]. These findings suggest that NLRP6 attenuates inflammatory signaling by suppressing NF-κB and ERK activation, thereby limiting NLRP3 inflammasome priming (Fig. 3).

Taken together, the data concerning the crosstalk between NLRP1, NLRP6, and NLRP3 reveal both shared and distinct regulatory principles. At the level of inflammasome priming, both NLRP1 and NLRP6 modulate NF-κB signaling, albeit in different ways: NLRP1 exerts context-dependent effects on NF-κB activity in a cell type-specific manner, whereas NLRP6 acts as a broadly negative regulator of NF-κB activity. At the level of inflammasome activation, lysosomal damage represents a convergence point for both NLRP6 and NLRP3, although whether they employ identical downstream mechanisms remains unclear. K⁺ efflux presents another point of potential convergence: while it is a well-established signal for NLRP3 activation, it also triggers NLRP1 activation in human keratinocytes through the ZAKα–ribotoxic stress pathway, raising the question of whether these two sensors are coactivated by the same stimulus and whether they synergize during infection. How these mechanistically distinct sensors coordinate their activities during complex infections involving multiple PAMPs and DAMPs is an important open question.

#### Crosstalk between NLRP11 and the NLRP3 inflammasome

NLRP11 (also known as NALP11, PYPAF6, and PAN10) is a primate-specific NLR that is absent in mice [235]. NLRP11 was initially identified as a negative regulator of type I interferon and NF-κB signaling [236–238], suggesting that it may act as a repressor of NLRP3 priming. More recent studies, however, have demonstrated that NLRP11 also participates in both canonical [239–241] and noncanonical inflammasome signaling in human macrophages [241, 242]. In the canonical pathway, NLRP11 was recruited to ASC specks together with NLRP3 following nigericin stimulation in THP-1 cells [239]. Genetic deletion of NLRP11 in THP-1 cells, primary human macrophages, and reconstituted HEK293T cells markedly impaired NLRP3 inflammasome-mediated caspase-1 activation and cytokine secretion in response to nigericin, ATP, and silica [239, 240]. Mechanistically, NLRP11 promoted ASC oligomerization through its PYD, thereby facilitating NLRP3 inflammasome assembly [239]. In addition, NLRP11 enhanced IL-1β release driven by CAPS-associated NLRP3 mutants [239]. These findings suggest that NLRP11 functions as a licensing factor for efficient NLRP3 inflammasome assembly (Fig. 3). Because NLRP11 is restricted to primates, an important future direction will be to determine how the NLRP3 inflammasome in nonprimate species, including mice, achieve efficient inflammasome assembly in the absence of NLRP11.

In parallel, NLRP11 has been implicated in noncanonical inflammasome activation. Cytosolic delivery of LPS, either by electroporation or during infection with gram-negative bacteria, induced caspase-4 cleavage and pyroptotic cell death, both of which were significantly reduced in NLRP11-deficient THP-1 cells [242]. Similarly, caspase-1 activation driven by LPS-induced noncanonical NLRP3 inflammasome signaling was also diminished in these cells [242]. Mechanistically, NLRP11 formed a complex with LPS and caspase-4 but not with caspase-5, the closely related human paralog of caspase-4 [242]. Because both NLRP11 and caspase-4 bound to LPS independently [242], NLRP11 has been proposed to function as an accessory cytosolic LPS sensor that enhances the sensitivity of human macrophages to gram-negative bacterial infection.

#### Crosstalk between NLRP12 and the NLRP3 inflammasome

In the NLR family, NLRP12 (also known as NALP12, MONARCH-1, and PYPAF7) is most closely related to NLRP3 according to molecular phylogenetic analyses [243]. Like NLRP3, NLRP12 contains an N-terminal PYD, a central NACHT, and a C-terminal LRR domain [244]. NLRP12 is highly expressed in granulocytes, including neutrophils and eosinophils, although its expression in monocytes and macrophages has shown conflicting results [243–245].

NLRP12 was initially identified as a positive regulator of NF-κB signaling. Coexpression of NLRP12 and ASC in HEK293T cells promoted NF-κB activation in a manner dependent on their interaction [244]. However, subsequent studies provided opposing evidence, suggesting a more prominent role for NLRP12 in suppressing both canonical and noncanonical NF-κB signaling [246–249]. In canonical NF-κB signaling, NLRP12 interacted with IRAK1 via its NACHT domain and inhibits IRAK1 autophosphorylation, thereby suppressing inflammatory responses induced by ligands of the TLR2, TLR4, and TNF receptor (TNFR) signaling pathways in reconstituted HEK293T cells, macrophages, and monocytes [247]. Notably, TLR agonists and TNF stimulation reduced NLRP12 expression [247], suggesting a mechanism that relieves this inhibitory brake during inflammatory signal activation. In noncanonical NF-κB signaling, NLRP12 interacted with NF-κB-inducing kinase (NIK) through its NACHT domain and LRR region, promoting NIK degradation via the proteasome [249]. In addition, NLRP12 was associated with and stabilizes TRAF3, thereby facilitating NIK degradation [246]. Consistent with its role in restraining inflammation, NLRP12-deficient mice were highly susceptible to colonic inflammation and tumorigenesis, accompanied by increased proinflammatory cytokine and chemokine production due to hyperactivation of NF-κB and ERK signaling in macrophages [250]. NLRP12 deficiency was also associated with resistance to *S*. Typhimurium infection [251]. *S*. Typhimurium infection induced NLRP12-mediated suppression of IκΒα and ERK phosphorylation, thereby limiting the production of inflammatory cytokines in the NF-κB and MAPK pathways [251]. Furthermore, loss-of-function mutations in human NLRP12 were associated with hereditary autoinflammatory syndromes characterized by recurrent fever and elevated cytokine production due to dysregulated NF-κB activation [252]. Given that NF-κB signaling provides a key priming signal for NLRP3 inflammasome activation, NLRP12 may indirectly regulate NLRP3 inflammasome activity through its control of upstream inflammatory signaling pathways (Fig. 3).

In addition to its role in regulating NF-κB signaling, recent studies have suggested that NLRP12 can also initiate inflammasome assembly and drive caspase-1 activation and IL-1β maturation (Fig. 3). Multiple human pathogens, including the bacteria *Yersinia pestis* [253], *S*. Typhimurium, *Klebsiella pneumoniae* [254], *Mycobacterium tuberculosis* [254], the positive-sense ssRNA virus Dengue Virus [255], and the parasite *Plasmodium chabaudi* [256], have been reported to stimulate NLRP12-associated responses. Compared with wild-type mice, both *Nlrp12*^−/−^ mice and *Nlrp3*^−/−^ mice exhibited reduced survival during infection with *Y. pestis*, with *Nlrp12*^−/−^ mice exhibiting a more severe phenotype [253], suggesting that *Y. pestis* infection activated both NLRP12 and the NLRP3 inflammasome, with NLRP12 playing a more prominent protective role. Surprisingly, *Y. pestis*-induced NF-κB signaling was not altered by NLRP12 deficiency in BMDMs [253]. Thus, NLRP12 contributes to inflammasome activation independent of NF-κB regulation in the context of *Y. pestis* infection. The upstream signals governing these effects remain incompletely understood. Disruption of the *Y. pestis* T3SS impaired NLRP12 inflammasome activation [253], suggesting that NLRP12 may sense a secreted bacterial effector, although the identity of a bona fide ligand remains unresolved.

Another example of synergy between NLRP3 and NLRP12 is infection with *P. chabaudi*, an experimental model of malaria. *P. chabaudi* infection in mice induced robust caspase-1 cleavage and IL-1β secretion, which were abolished in both *Nlrp12*^−/−^ mice and *Nlrp3*^−/−^ mice [256]. Surprisingly, P2X_7_ was required for this activity, indicating that *P. chabaudi* infection may induce ATP release to engage the P2X_7_–NLRP3 axis. How NLRP3 and NLRP12 cooperate during *P. chabaudi* infection to drive inflammation is still unknown. However, NLRP3 specks and NLRP12 specks in *P. chabaudi*-infected peripheral blood mononuclear cells (PBMCs) did not colocalize [256], suggesting that these two sensors form distinct inflammasome complexes. In contrast, a recent study proposed that NLRP12 may associate with NLRP3 and ASC within a single higher-order signaling platform termed the PANoptosome [257]. NLRC5, another NLR family member highly expressed in hematopoietic cells, was reported to promote the formation of this PANoptosome complex [258]. Heme, a DAMP released from damaged or dying red blood cells, was shown to induce caspase-1 activation and IL-1β secretion in BMDMs in the presence of a priming signal, such as the TLR1/2 ligand Pam3CSK4 [257]. These responses are dependent on both NLRP3 and NLRP12 [257]. Immunoprecipitation of ASC pulled down NLRP3 in an NLRP12-dependent manner [257], supporting the idea that these proteins were in the same signaling complex. Notably, heme-induced NLRP12–NLRP3 inflammasome activation required prolonged stimulation (36–48 h) [257, 258], suggesting slow assembly kinetics. A remaining question is the temporal dynamics of PANoptosome formation, which will require dedicated time-course analyses.

In addition to the positive regulatory role of NLRP12 in inflammasome signaling discussed above, another study reported that NLRP12 can also act as a negative regulator of the NLRP3 inflammasome pathway in a species-specific manner [259]. Overexpression of human NLRP12 in HEK293T cells reduced ASC speck formation driven by both human wild-type NLRP3 and CAPS-associated human NLRP3 mutants. In contrast, murine NLRP12 only reduced ASC assembly driven by murine CAPS-associated NLRP3 mutants but had little effect on murine wild-type NLRP3. The PYD of NLRP12 was dispensable for this inhibitory effect [259], suggesting that NLRP12 suppresses NLRP3 signaling through direct interaction rather than by competing for ASC recruitment. The clinical relevance of this inhibitory function was supported by observations that monocytes from two patients carrying an NLRP12 nonsense mutation exhibited enhanced NLRP3 signaling compared with those from an unaffected family member [259]. Consistent with previous findings that NLRP12 had a lower capacity to promote ASC polymerization than NLRP3 and NLRP6 do [243, 259], NLRP12 is increasingly considered a multifunctional immune regulator. Its precise role in inflammasome outcomes likely depends on the species, cell types, and nature of the activating stimulus.

#### Crosstalk between AIM2 and the NLRP3 inflammasome

AIM2 is a hematopoietic interferon-inducible nuclear protein with a 200 amino acid repeat (HIN200) protein family member that detects dsDNA in the cytosol, a signal indicative of microbial invasion, cellular damage, or genomic instability [260–263]. AIM2 contains an N-terminal PYD and a C-terminal HIN-200 domain. In its resting state, the HIN-200 domain engages the PYD in an autoinhibitory interaction. Cytosolic DNA binds to the positively charged HIN-200 domain in a sequence-independent but length-dependent manner [264, 265]. This binding releases autoinhibition and promotes AIM2 oligomerization along the dsDNA scaffold [264]. Multiple AIM2 molecules coassemble cooperatively, forming a filamentous platform that leads to the formation of inflammasome complexes [266–268]. In infections, AIM2 detects dsDNA from bacteria, viruses, and parasites, driving protective inflammatory responses [269–275]. However, dysregulated AIM2 activity contributes to autoinflammatory and autoimmune diseases, including systemic lupus erythematosus (SLE), in which self-dsDNA aberrantly activates the sensor [276, 277]. In cancer, AIM2 plays a complex, context-dependent role, acting as a tumor suppressor in some settings, such as colorectal [278–280] and breast cancer [281], while promoting inflammation-driven tumor progression in others [282–284].

AIM2 and the NLRP3 inflammasome can be activated concurrently during infection, as many bacterial, viral, and eukaryotic pathogens harbor multiple PAMPs capable of stimulating both sensors simultaneously. *Aspergillus fumigatus*, a fungus that causes invasive pulmonary aspergillosis in immunocompromised individuals, provides one such example. *A. fumigatus* infection activated both AIM2 and NLRP3, and mice deficient in either sensor showed heightened susceptibility to invasive aspergillosis [285]. A second example is *Plasmodium falciparum*, the protozoan parasite responsible for life-threatening malaria. During replication inside red blood cells, *P. falciparum* degraded hemoglobin, leading to the accumulation of heme, which was subsequently polymerized into hemozoin (Hz), an inert crystal. Hz primed the NLRP3 pathway by activating TLR9 signaling [286] before acting as a direct NLRP3 stimulus, although the precise mechanism is unclear [272]. Concurrently, Hz destabilized phagolysosomes, releasing luminal contents that were sensed by AIM2, thereby engaging both inflammasome pathways during a single infection [272].

In some cases, the ligands for both NLRP3 and AIM2 inflammasomes are unmasked by the same host proteins. Upon internalization by gram-negative bacteria, guanylate-binding proteins (GBPs) were recruited to the bacterial surface [271, 287, 288] and facilitated the subsequent targeting of IRGB10 [289]. IRGB10 disrupted the bacterial envelope, liberating cytosolic bacterial DNA and LPS, which activated the AIM2 inflammasome and the noncanonical NLRP3 inflammasome, respectively [289]. This coordinated unmasking serves two potential purposes: it amplifies inflammatory signaling by engaging two inflammasome pathways simultaneously, and it provides a fail-safe mechanism when one pathway is evaded. For example, *F. novicida* bears tetraacylated LPS that evaded murine caspase-11 detection, thereby escaping noncanonical NLRP3 activation [290]; however, the IRGB10-mediated release of bacterial DNA still engaged the host AIM2 inflammasome, ensuring an effective innate immune response [289].

### The Nlrp3 inflammasome and cell death pathways

Multicellular organisms encode a variety of cell death pathways that play essential roles in tissue development, maintenance of cellular homeostasis, regulation of inflammation, elimination of infected cells, and defense against cancer [291]. Among the best-characterized types of programmed cell death pathways are apoptosis, necroptosis, and pyroptosis [292]. The suppression of a particular cell death pathway may facilitate the activation of alternative death programs, an evolutionary adaptation thought to preserve host defense in the face of pathogen-mediated blockade of host cell death signaling [293]. Although these three pathways share the common outcome of cellular destruction, they differ substantially in their upstream triggers, signaling mechanisms, execution machinery, and immunological consequences.

Apoptosis is generally considered a nonlytic form of cell death that preserves plasma membrane integrity, whereas necroptosis and pyroptosis are lytic processes associated with membrane rupture and the release of intracellular contents [292]. Apoptosis in mammalian cells occurs through two signaling pathways: intrinsic apoptosis triggered by intracellular stress signals and extrinsic apoptosis triggered by the activation of cell-surface death receptors. Intrinsic apoptosis is initiated by the sensing of environmental changes, including DNA damage, ER stress, and growth factor withdrawal [294]. Under normal conditions, apoptosis is suppressed by pro-survival members of the B-cell lymphoma 2 (BCL-2) family, such as BCL-2, BCL-xL, MCL-1, and BCL-W [295]. In intrinsic apoptosis, cellular stress signals activate BH3-only proteins, such as BID, BIM, PUMA, NOXA, and BAD [291], which neutralize pro-surviving BCL-2 family members. This relieves the inhibition of BCL-2 associated X (BAX) and BCL-2 antagonist/killer 1 (BAK) [296]. BAX and BAK oligomerize within the mitochondrial outer membrane and drive mitochondrial outer membrane permeabilization (MOMP) [297]. Subsequently, cytochrome c and second mitochondria-derived activator of caspase (SMAC, also known as DIABLO) are released through the ruptured outer mitochondrial membrane into the cytosol [298]. Cytochrome c binds to the adaptor protein apoptotic peptidase activating factor 1 (APAF-1) [299] and promotes its oligomerization into a supramolecular complex known as the apoptosome [300]. The apoptosome mediates the activation of caspase-9, an initiator caspase [301]. Activated caspase-9 then proteolytically processes executioner caspases, including caspase-3 and caspase-7 [302]. Once activated, caspase-3 and caspase-7 cleave hundreds of downstream substrate proteins. Cleavage of these substrates mediates the morphological and biochemical hallmarks of apoptosis, including DNA fragmentation, nuclear condensation, phosphatidylserine (PS) exposure, membrane blebbing, and apoptotic body formation [294].

In contrast, extrinsic apoptosis is initiated by transmembrane death receptors harboring a protein–protein interaction domain known as the death domain, which are activated by their cognate extracellular ligands [303]. Examples include Fas ligand (FasL) binding to Fas (also known as CD95) [304], TNF binding to TNF receptor 1 (TNFR1), and TNF-related apoptosis-inducing ligand (TRAIL) binding to TRAIL-R1 (also known as DR4) and TRAIL-R2 (also known as DR5) [291]. Ligand-induced receptor oligomerization promotes the recruitment of adaptor proteins containing death domains, such as Fas-associated death domain protein (FADD) and TNF receptor type 1-associated death domain protein (TRADD). The adaptor proteins recruit caspase-8, an initiator caspase, through death effector domain interactions. Like caspase-9 in intrinsic apoptosis, caspase-8 in extrinsic apoptosis directly cleaves executioner caspases, including caspase-3 and caspase-7. Caspase-8 also amplifies apoptotic signaling through cleavage of BID into truncated BID (tBID), which is translocated to mitochondria and promotes BAX/BAK activation. Consequently, the intrinsic and extrinsic apoptotic pathways are closely linked and can reinforce one another depending on the cellular context.

While Fas signaling predominantly induces apoptosis, TNF signaling can result in either pro-survival or pro-apoptotic outcomes depending on how the receptor-associated signaling complex is assembled and regulated over time [305–307]. Upon TNF binding, TNFR1 first forms a membrane-bound signaling complex known as complex I [308], which consists of TRADD, receptor-interacting protein kinase 1 (RIPK1) [309], the ubiquitin ligases TRAF2/5, cellular inhibitor of apoptosis proteins (cIAP)1/2 [310–312], and linear ubiquitin chain assembly complex (LUBAC) [313]. Within complex I, RIPK1 is ubiquitinated by cIAP1/2 and LUBAC and recruits the canonical IKK complex to activate the pro-survival and proinflammatory NF-κB and MAPK signaling pathways. NF-κB signaling also drives the expression of X-linked inhibitor of apoptosis (XIAP), which inhibits the functions of caspases such as caspase-3 and caspase-7, further promoting survival. A switch from pro-survival signaling to cell death occurs when ubiquitin-dependent signaling is disrupted [292], e.g., by inhibition or loss of cIAPs or by the translation inhibitor cycloheximide-mediated inhibition of NF-κB signaling [314]. Under these conditions, TNF signaling transitions from membrane-associated complex I to cytosolic complex II [308]. Within complex II, caspase-8 initiates the extrinsic apoptotic cascade [315].

Necroptosis is initiated when extrinsic apoptotic signaling is inhibited, most commonly through the loss or inhibition of caspase-8. As a result, necroptosis shares several upstream components with the extrinsic apoptosis pathway. In addition to death receptors, necroptosis can also be activated by PRRs, including TLR3, TLR4, or Z-DNA binding protein 1 (ZBP1, also known as DAI) [316, 317]. When caspase-8 is activated, it proteolytically cleaves RIPK1 to suppress its kinase activity, acting as a brake on necroptosis. In the absence of caspase-8 activity, uncleaved RIPK1 interacts with receptor-interacting protein kinase 3 (RIPK3) through its RIP homotypic interaction motifs (RHIMs) to mediate RIPK3 phosphorylation, resulting in the formation of a necroptotic signaling complex known as the necrosome [318–321]. Within the necrosome, RIPK3 phosphorylates mixed lineage kinase domain-like protein (MLKL) to promote its activation [322, 323]. MLKL then translocates to the plasma membrane to form pores and drive necroptosis [323]. It has been proposed that necroptosis evolved as a host defense mechanism against viruses that suppress apoptosis by inhibiting caspase-8 [291]. Thus, caspase-8 functions not only as an initiator of extrinsic apoptosis but also as a suppressor of necroptosis.

While the NLRP3 inflammasome primarily drives pyroptotic cell death through caspase-1 activation and GSDMD cleavage, its signaling components and downstream effects are extensively interconnected with those of other cell death pathways, including apoptosis and necroptosis, as discussed below.

#### Caspase-8 and FADD in NLRP3 priming

Caspase-8 is traditionally regarded as a key component of the pro-death arm of extrinsic apoptosis. However, several early studies have also implicated caspase-8 in the prosurvival arm. Overexpression of caspase-8 activated NF-κB signaling in HEK293 cells independent of its apoptotic function [324]. Furthermore, caspase-8 was required for NF-κB activation induced by overexpression of c-FLIP, an antiapoptotic regulator, in HEK293 cells [325]. Consistent with these findings, caspase-8 deficiency in B cells attenuated NF-κB signaling downstream of TLR2, TLR3, and TLR4, suggesting that caspase-8 acts as a positive regulator of the NF-κB pathway [326]. Although the precise mechanisms by which caspase-8 promotes NF-κB signaling remain unclear, caspase-8 has been found in a complex containing the IKK complex [326], indicating that it may directly modulate IKK kinase activity. This activity facilitated the phosphorylation of IκBα, an inhibitor that sequesters NF-κB in the cytosol, thereby promoting NF-κB activation.

Given the critical role of NF-κB signaling in providing the priming signal for NLRP3 inflammasome activation, subsequent studies investigated the contributions of caspase-8 and other apoptotic components to this process. Both caspase-8-deficient and FADD-deficient mice exhibited embryonic lethality because of RIPK3-mediated necroptosis during development [327–330]. To circumvent this limitation while studying the roles of caspase-8 and FADD in the NLRP3 inflammasome, bone marrow-derived macrophages (BMDMs) from caspase-8/RIPK3 double-deficient mice were compared with those from RIPK3-deficient controls [331]. Whereas RIPK3 deficiency alone did not affect canonical NLRP3 inflammasome activation induced by nigericin, ATP, or noncanonical NLRP3 inflammasome activation induced by infection with the gram-negative bacteria *Citrobacter rodentium* or *Escherichia coli*, combined deficiency of caspase-8/RIPK3 or FADD/RIPK3 markedly impaired caspase-1 cleavage [331]. In contrast, neither deficiency affected caspase-1 cleavage following infection with *S*. Typhimurium [331], which predominantly activated the NAIP–NLRC4 inflammasome [188]. The effects of caspase-8 and FADD on NLRP3 inflammasome activation appear to be mediated, at least in part, through their roles in priming. *Ripk3*^−/−^*Fadd*^−/−^ BMDMs exhibited impaired LPS-induced expression of NLRP3 and pro-IL-1β [331]. The importance of caspase-8 in TLR-mediated priming was further supported by an independent study, which revealed that *Ripk3*^−/−^*Casp8*^−/−^ BMDMs produced lower levels of pro-IL-1β following LPS stimulation [48] (Table 1). Consequently, these cells displayed markedly reduced IL-1β secretion in response to subsequent treatment with nigericin, ATP, or alum [48].

**Fig. 4:**
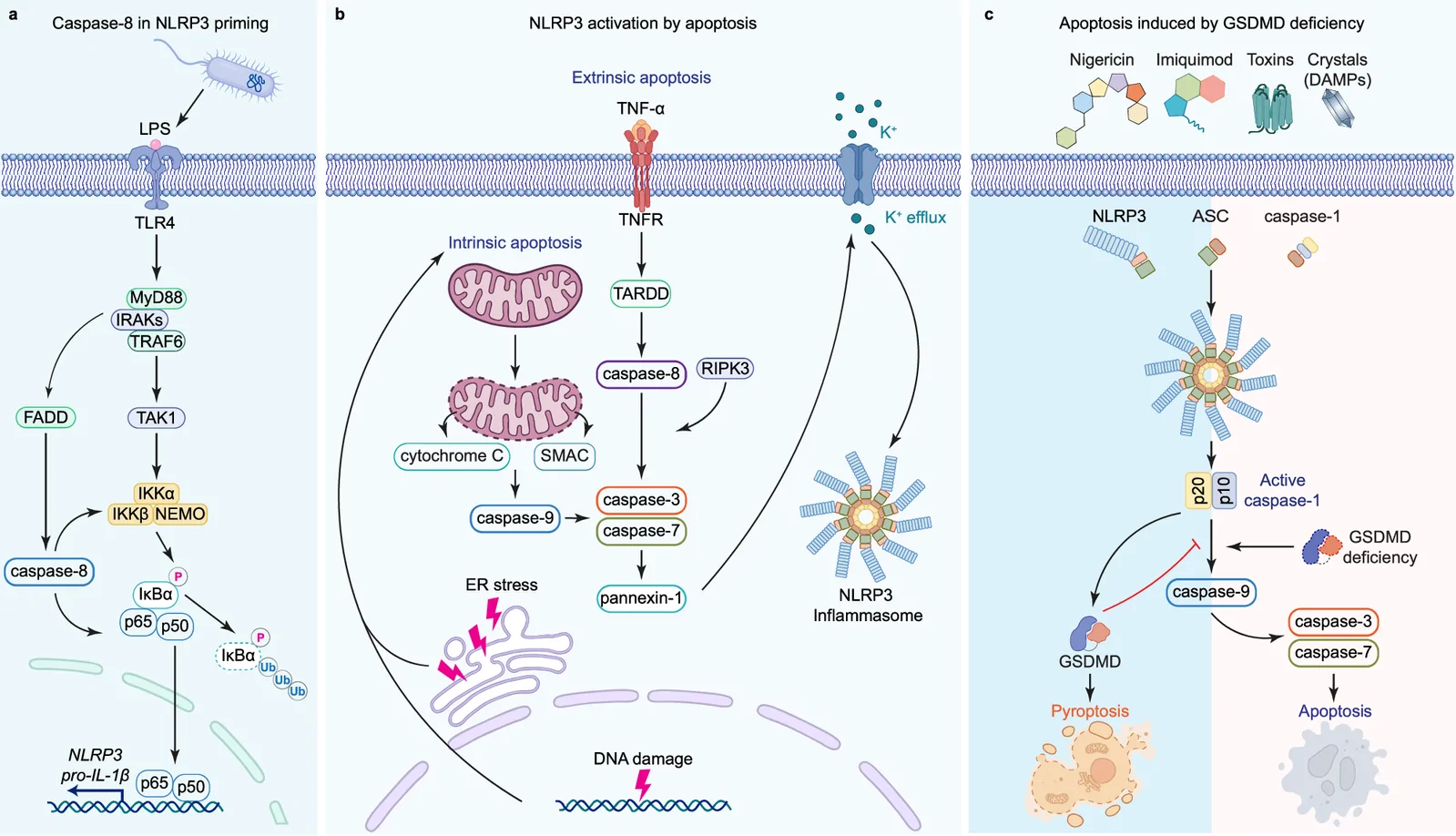
Crosstalk between the NLRP3 inflammasome and cell death pathways. **a** Proposed roles of caspase-8 in NLRP3 priming. Fas-associated death domain protein (FADD) and caspase-8 are activated downstream of Toll-like receptor (TLR) signaling to promote NLRP3 priming. Although the precise mechanisms remain incompletely understood, caspase-8 may facilitate priming by promoting IKK complex activation or by acting downstream of IκBα degradation, thereby increasing the expression of NLRP3 and pro-IL-1β. **b** Proposed mechanism of NLRP3 inflammasome activation by apoptosis. Both intrinsic and extrinsic apoptotic pathways can activate the NLRP3 inflammasome, whether initiated by infectious agents or sterile conditions. Intrinsic apoptosis is initiated by cellular stress signals, such as DNA damage and endoplasmic reticulum (ER) stress. Cellular stress induces mitochondrial outer membrane permeabilization (MOMP), resulting in the release of cytochrome c and second mitochondria-derived activator of caspases (SMAC) into the cytosol and subsequent activation of caspase-9. In extrinsic apoptosis, tumor necrosis factor (TNF) signaling promotes the formation of a signaling complex containing TNF receptor type 1-associated DEATH domain protein (TRADD), leading to caspase-8 activation. Caspase-8 and caspase-9 subsequently activate the executioner caspases, caspase-3 and caspase-7, which cleave and activate pannexin-1. Activated pannexin-1 forms plasma membrane channels that promote potassium (K^+^) efflux, thereby triggering NLRP3 inflammasome activation. **c** Proposed mechanism of apoptosis induced by GSDMD deficiency. Assembly of the NLRP3 inflammasome activates caspase-1, which cleaves GSDMD to generate the pore-forming N-terminal fragment that mediates pyroptosis. In addition to driving pyroptotic cell death, GSDMD activation suppresses caspase-9-mediated apoptotic signaling. In contrast, when GSDMD is absent or inhibited, caspase-9 activity is relieved, allowing it to activate caspase-3 and caspase-7, thereby shifting the cell fate from pyroptosis to apoptosis. DAMPs, damage-associated molecular patterns

Together, these findings indicate that caspase-8 and FADD contribute to NLRP3 inflammasome activation by promoting the transcriptional priming events required for robust inflammasome responses (Fig. 4a). However, the molecular mechanisms by which caspase-8 and FADD promote the priming step have not been fully resolved. One study reported that the LPS-induced phosphorylation of IκBα and ERK was reduced in *Ripk3*^−/−^*Casp8*^−/−^ BMDMs[331], suggesting that caspase-8 functions upstream of IKK-mediated IκBα phosphorylation. In contrast, another study revealed no defects in LPS-induced IκBα degradation or p65 phosphorylation in *Ripk3*^−/−^*Casp8*^−/−^ BMDMs, both of which are downstream of IκBα phosphorylation [48]. It therefore remains unclear whether caspase-8 acts upstream of IκBα phosphorylation and degradation, potentially through regulation of IKK complex activity, or instead targets a more downstream event to promote NLRP3 and pro-IL-1β expression.

Another study [332] revealed that the requirement for caspase-8 during NLRP3 inflammasome priming depends on both the priming stimulus and the duration of priming. *Ripk3*^−/−^*Casp8*^−/−^ and *Ripk3*^−/−^*Fadd*^−/−^ BMDMs exhibited robust caspase-1 cleavage when primed for a short period (10 min) with either the TLR4 ligand LPS or the TLR1/2 ligand Pam3CSK4 prior to ATP stimulation. However, prolonged LPS priming (3 h) resulted in a partial defect in caspase-1 cleavage in these cells [332]. In contrast, compared with *Ripk3*^−/−^ BMDMs, *Ripk3*^−/−^*Casp8*^−/−^ and *Ripk3*^−/−^*Fadd*^−/−^ BMDMs displayed markedly impaired caspase-1 cleavage following priming with the TLR3 ligand poly(I:C) and subsequent ATP treatment, regardless of the duration of priming [332]. These findings suggest that caspase-8 and FADD contribute to the transcription-dependent priming of NLRP3 inflammasome activation but are largely dispensable for transcription-independent priming. In addition, TLR3 may preferentially engage in transcription-dependent priming pathways and is therefore more sensitive to the loss of caspase-8 and FADD. Although the underlying mechanisms remain unclear, differences in adaptor usage among TLR signaling pathways [333] and the kinetics of signaling complex assembly may account for this context-dependent requirement.

Interestingly, stimulation of BMDMs with LPS followed by canonical NLRP3 activators, such as ATP, induced caspase-8 cleavage in a manner dependent on NLRP3 and ASC [331]. In contrast, noncanonical inflammasome stimuli, such as infection with *C. rodentium* or *E. coli*, also triggered caspase-8 cleavage, but this process occurred independently of NLRP3 and ASC [331]. Thus, canonical and noncanonical NLRP3 stimuli engage distinct pathways to promote caspase-8 activation. It remains to be elucidated whether caspase-11/4/5 are involved in caspase-8 cleavage in response to noncanonical stimuli. Nevertheless, for both types of stimuli, the resulting caspase-8 activation may further enhance priming signals, thereby establishing a positive-feedback loop that amplifies inflammasome activation.

The key roles of caspase-8 and FADD in NLRP3 inflammasome priming were further validated in vivo. In an LPS-induced endotoxemia model, the circulating levels of IL-1β and IL-1α were significantly lower in *Ripk3*^−/−^*Casp8*^−/−^ and *Ripk3*^−/−^*Fadd*^−/−^ mice than in *Ripk3*^−/−^ control mice following LPS challenge [331]. Consistent with these findings, compared with Ripk3^−/−^ mice, *Ripk3*^−/−^*Casp8*^−/−^ and *Ripk3*^−/−^*Fadd*^−/−^ mice infected with *C. rodentium* presented increased bacterial burdens and more severe disease [331]. These results demonstrate that caspase-8 and FADD contribute to host defense against bacterial infection, at least in part, by promoting NLRP3 inflammasome priming and the subsequent production of proinflammatory cytokines.

#### NLRP3 inflammasome activation downstream of apoptosis

While caspase-8 contributed to the priming step, it was not required for NLRP3 inflammasome activation by nigericin or ATP [334]. Similarly, RIPK3 was dispensable for NLRP3 inflammasome activation induced by canonical stimuli, including nigericin, ATP, alum, and imiquimod [334, 335]. However, the NLRP3 inflammasome can also be activated downstream of apoptosis or necroptosis. In this context, these cell death pathways provide signal 2, which engages the NLRP3 inflammasome (Fig. 4b).

Both intrinsic and extrinsic apoptotic pathways can activate the NLRP3 inflammasome. BH3 mimetics are anticancer agents developed to inhibit BCL-2 family proteins, including ABT-737, which targets BCL-2, BCL-xL, and BCL-W [336, 337], and S63845, which targets MCL-1 [338]. These compounds are typically used to induce intrinsic apoptosis. In contrast, SMAC mimetics, such as AZD5582, mimic SMAC, a mitochondrial protein released during apoptosis that antagonizes inhibitor of apoptosis proteins (IAPs) [339]. In parallel, inhibitors of TAK1, such as 5z-7-oxozeaenol, suppress TNF-induced pro-survival NF-κB and MAPK signaling, thereby sensitizing cells to apoptosis [96]. Accordingly, SMAC mimetics and TAK1 inhibitors are often combined with TNF to induce extrinsic apoptosis. Notably, the induction of either intrinsic or extrinsic apoptosis in BMDMs using these approaches led to NLRP3-dependent caspase-1 activation [27]. Deletion of A1, a pro-survival BCL-2 family member, further promoted NLRP3 inflammasome activation induced by intrinsic apoptosis after LPS priming [340]. One intriguing observation was that depletion of RIPK3 markedly reduced caspase-1 cleavage induced by both intrinsic and extrinsic apoptosis [27]. RIPK3 is a key molecular component of necroptosis but is not a major physiological regulator of apoptosis [292]. Indeed, neither RIPK3 kinase activity nor MLKL activity was required for caspase-1 activation downstream of apoptosis [27]. Thus, how apoptotic signaling promotes NLRP3 inflammasome activation remains to be determined.

Despite robust cleavage of GSDMD under these conditions, it was not required for caspase-1 activation [27]. In contrast, depletion of pannexin-1 markedly reduced caspase-1 cleavage induced by both apoptotic pathways. Pannexin-1 is a plasma membrane channel protein that can be cleaved by caspase-3 and caspase-7 and has been shown to facilitate the clearance of apoptotic cells [341, 342]. These findings suggest that NLRP3 activation downstream of apoptosis is driven by membrane permeabilization caused by pannexin-1 rather than by GSDMD. Because pores formed by both pannexin-1 and GSDMD can, in principle, promote K^+^ efflux, future studies are needed to determine how these pores differ in their biophysical properties and whether they differentially regulate the release of intracellular metabolites, such as ATP, that contribute to NLRP3 inflammasome activation.

#### NLRP3 inflammasome activation downstream of necroptosis

In addition to apoptosis, necroptosis has been reported to activate the NLRP3 inflammasome. Compound A (Cp. A) is a SMAC mimetic that antagonizes all three IAPs. Combined treatment with Cp. A, LPS and a low dose of the pancaspase inhibitor Q-VD-OPh (QVD), which inhibits apoptotic and necroptotic caspases but not caspase-1, were used to induce necroptosis in BMDMs. This treatment led to caspase-1 activation and IL-1β maturation in a manner dependent on NLRP3, RIPK3, and MLKL [343], suggesting that necroptosis activates the NLRP3 inflammasome. Consistent with previous results [48, 331], RIPK3 or MLKL deficiency did not affect LPS-induced pro-IL-1β priming [343], indicating that these molecules act at the activation rather than the priming stage. Necroptotic cell death under these conditions was dependent on RIPK3 and MLKL but independent of NLRP3 and caspase-1 [343]. In addition, the combined treatment induced MLKL oligomerization and translocation to the plasma membrane, which resulted in K⁺ efflux that was required for caspase-1 activation and IL-1β maturation, indicating that K⁺ efflux is a key mechanism through which necroptosis activates the NLRP3 inflammasome.

GSDMD is required for IL-1β secretion in response to multiple microbial and self-derived inflammasome stimuli [16–18, 344]. Surprisingly, IL-1β release induced by LPS/Cp. A/QVD-mediated necroptosis was independent of GSDMD [343]. One possibility is that MLKL-mediated plasma membrane permeabilization is sufficient for IL-1β secretion. This model was supported by the observation that inducible expression of a constitutively active MLKL mutant (S345D) induced necroptosis and IL-1β secretion in cells primed with the TLR1/2 ligand Pam3CSK4 [343]. Necroptosis has been implicated in a range of human diseases, including inflammatory bowel disease, ischemia–reperfusion injury, neurodegeneration, and sepsis, in which excessive or dysregulated inflammatory cell death contributes to tissue damage [345–347]. Activation of the NLRP3 inflammasome downstream of necroptosis may amplify these pathological processes by promoting IL-1β and IL-18 production in response to membrane rupture and ion flux. As a result, targeting necroptosis or its coupling to NLRP3 activation may represent a therapeutic strategy to limit inflammation-driven tissue injury under these conditions.

Necroptosis is a regulated, programmed form of necrotic cell death driven by defined signaling pathways such as RIPK1, RIPK3, and MLKL, whereas unregulated (or accidental) necrosis refers to cell death associated with the loss of membrane integrity and uncontrolled cellular breakdown in response to severe physical, chemical, or metabolic injury and proceeds without dedicated signaling machinery, leading to passive cell lysis and the release of intracellular contents [348]. While necroptosis-driven NLRP3 activation is cell intrinsic, stress-induced necrosis has been reported to activate NLRP3 through a cell-extrinsic mechanism. Necrosis induced by various stressors, including pressure overload, hypoxic injury, and complement-mediated damage, led to plasma membrane rupture and the release of intracellular contents that promoted NLRP3 inflammasome activation in neighboring cells and contributed to renal ischemic acute tubular necrosis in vivo [349]. ATP has been proposed as a candidate mediator of this effect, although this hypothesis requires further testing in P2X_7_-deficient cells.

#### NLRP3 inflammasome activation in caspase-8-deficient dendritic cells

Another example of NLRP3 inflammasome activation linked to necroptosis-related signaling is observed in caspase-8-deficient dendritic cells. As noted above, caspase-8 deficiency in macrophages such as BMDMs impaired NLRP3 priming [48, 331]. In contrast, caspase-8 deficiency in dendritic cells did not affect IL-1β activation in response to ATP [350], suggesting that caspase-8 is not essential for either the priming or activation of the canonical NLRP3 inflammasome in this cell type. Instead, caspase-8 deficiency in dendritic cells enabled LPS priming alone to trigger NLRP3 activation [350] (Table 1). This response was dependent on RIPK1, RIPK3, and MLKL, which is consistent with the involvement of necroptosis-related signaling and the formation of a RIPK1–RIPK3–FADD-containing necrosome in the absence of caspase-8 [350]. Because caspase-8 deficiency in dendritic cells did not increase cell death compared with that in wild-type cells [350], NLRP3 activation was unlikely to be driven by the passive release of intracellular contents or K^+^ efflux alone. This finding was supported by the observation that cell extracts from LPS-treated caspase-8-deficient dendritic cells failed to activate the NLRP3 inflammasome when they were transferred to other cells [350]. In addition, neither ROS production nor ATP accumulation was significantly altered in caspase-8-deficient dendritic cells. Thus, the precise cell-autonomous mechanisms linking caspase-8 deficiency to NLRP3 activation remain unclear.

A related finding was the identification of a caspase-8 mutation that induced inflammasome activation during development. Caspase-8 functions as the initiator caspase in extrinsic apoptosis [351, 352] while also acting as an inhibitor of necroptosis by suppressing RIPK3–MLKL signaling [292]. *Casp8*^−/−^ mice exhibited embryonic lethality because of uncontrolled necroptosis, which could be rescued by the deletion of RIPK3 or MLKL [330, 353, 354]. Surprisingly, mice carrying an enzymatically inactive caspase-8 C362S mutation (*Casp8*^C362S^/^C362S^) remained perinatally lethal even in the absence of MLKL [355], suggesting that caspase-8 functions independently of its catalytic activity. Indeed, the caspase-8 C362S mutation promoted ASC speck formation and caspase-1 and IL-1β activation both in vitro and in vivo. This inflammasome activity contributed to the perinatal lethality of *Casp8*^C362S^/^C362S^*Mlkl*^−/−^, as genetic deletion of ASC or caspase-1 significantly improved survival and reduced pathology [355]. Although this phenotype was proposed to involve NLRP3 inflammasome activation, further studies using *Casp8*^C362S^/^C362S^*Mlkl*^−/−^ mice crossed with *Nlrp3*^−/−^ mice or other inflammasome receptor-deficient mice are needed to define the specific inflammasome pathway involved.

NLRP3 inflammasome activation in response to LPS priming alone has also been reported in human monocytes [356]. This response was preserved in human monocytes with single deficiencies in RIPK1, RIPK3, FADD, or caspase-8 but was abolished in cells with combined deficiencies of RIPK1/RIPK3, FADD/RIPK3, caspase-8/RIPK3, or MLKL/caspase-8 [356]. These findings suggest that redundant or compensatory interactions among necroptosis-related components are required for this form of inflammasome activation. Further studies are needed to determine whether the mechanisms underlying this response in human monocytes are shared, at least in part, with those described in mouse dendritic cells.

#### IAP loss promotes caspase-8-mediated NLRP3 activation

Deficiencies in other apoptosis- and necroptosis-related components also facilitate NLRP3 inflammasome activation. One of the best characterized examples is the loss of IAPs, key regulators of apoptosis and necroptosis. Simultaneous inhibition of multiple IAPs, including cIAP1, cIAP2, and XIAP, either through genetic deletion or pharmacological inhibition, promoted robust NLRP3-dependent caspase-1 activation and IL-1β processing following LPS priming in BMDMs [335]. Notably, triple IAP-deficient (*Ciap1*^−/−^*Ciap2*^−/−^*Xiap*^−/−^) BMDMs, but not single- or double-deficient cells, displayed this activity [335]. Thus, cIAP1, cIAP2, and XIAP function redundantly to restrain NLRP3 inflammasome activation under basal conditions, and the loss of all three proteins is required to promote inflammasome activation in the presence of a priming signal. Surprisingly, although NLRP3 and ASC were required for caspase-1 activation in response to LPS in the absence of IAP activity, they were only partially responsible for IL-1β processing [335]. These findings suggested that IAP loss triggered both NLRP3–ASC-dependent and NLRP3–ASC-independent pathways for IL-1β maturation. Notably, depletion of caspase-8, but not caspase-2, caspase-3, caspase-7, or caspase-11, reduced IL-1β processing mediated by the NLRP3-independent pathway [335], indicating that caspase-8 was likely the protease responsible for IL-1β cleavage under these conditions.

In the absence of IAPs, TLR priming not only induced the expression of NLRP3 and pro-IL-1β but also promoted RIPK3 activation [334]. Both NLRP3-dependent and NLRP3-independent IL-1β processing required RIPK3 [335]. In contrast, the activation of the NLRP3 inflammasome by canonical stimuli, including nigericin, ATP, alum, and imiquimod, was independent of RIPK3 [335]. Although RIPK3 activation during necroptosis typically requires RIPK1 kinase activity [318–320], treatment with necrostatin-1, a RIPK1 kinase inhibitor [357, 358], did not inhibit the secretion of IL-1β induced by LPS in the absence of IAP activity [335]. Furthermore, caspase-8 activation in response to LPS in the absence of IAP activity occurred independently of RIPK3 kinase activity and MLKL [334]. These findings suggest that the loss of IAPs promotes both NLRP3 inflammasome activation and caspase-8 activity through mechanisms distinct from those involved in TNF-induced necroptosis. Although the pathway linking the loss of IAP-mediated RIPK3 activation to NLRP3 stimulation remains incompletely understood, available evidence suggests that RIPK3-dependent caspase-8 activation contributes to this process [334].

Similar to IAP deficiency, the loss of MCL-1 and BCL-xL, two pro-survival members of the BCL-2 family, also resulted in NLRP3-dependent caspase-1 activation in LPS-primed BMDMs [359]. This response required BAX, BAK, caspase-3, and caspase-7 and was associated with the loss of cIAP1 [359], suggesting that the underlying mechanism may overlap with that of IAP deficiency-induced NLRP3 activation. Consistent with these discoveries, another study reported that activation of TLR–MyD88 signaling promoted degradation of the E3 ubiquitin ligase TRAF2, leading to reduced cIAP1 levels and enhanced NLRP3 inflammasome activation [360]. These findings suggest that pathogen-derived signals and cellular stressors can decrease IAP activity, thereby promoting proinflammatory signaling through the NLRP3 inflammasome. In vivo, similar mechanisms have been linked to spontaneous inflammatory arthritis [334] and to inflammatory bowel disease associated with XIAP deficiency in human patients [361]. Together, these observations highlight the importance of IAPs in restraining inflammasome activation and maintaining immune homeostasis.

#### The NLRP3 inflammasome promotes apoptosis

While apoptosis can promote NLRP3 inflammasome activation, the NLRP3 inflammasome can also contribute to apoptotic signaling under certain conditions. One notable example is the crosstalk between extrinsic apoptosis and the NLRP3 inflammasome in epithelial cells (Table 1). In addition to macrophages and dendritic cells, NLRP3 inflammasome components are expressed in a variety of nonhematopoietic cell types [362], and cell type-specific differences can influence signaling mechanisms and biological outcomes [33, 356, 363]. In primary mouse tubular epithelial cells (TECs), treatment with TNFα and cycloheximide induced extrinsic apoptosis by inhibiting NF-κB-dependent pro-survival signaling. Surprisingly, caspase-8 and caspase-3 cleavage induced by this treatment was reduced in NLRP3-deficient and ASC-deficient cells [364]. In contrast, TNFα/cycloheximide-induced caspase-8 cleavage in peritoneal macrophages was unaffected by NLRP3 deficiency [364]. Thus, apoptotic signaling engaged the NLRP3 inflammasome to promote caspase-8 activation in epithelial cells but not in macrophages. This model is consistent with previous reports demonstrating that caspase-8 could be activated downstream of the NLRP3, NAIP–NLRC4, and AIM2 inflammasomes [182, 365–367].

The molecular basis for this cell type-specific difference remains unclear, and future studies are needed to determine whether epithelial cells express unique factors that promote NLRP3-dependent caspase-8 activation or, alternatively, lack inhibitory mechanisms present in macrophages and other cell types. Compared with macrophages, TECs express little, if any, caspase-1 [364]. Therefore, one possibility that the NLRP3 inflammasome facilitates caspase-8 activation in TECs is that caspase-1 deficiency allows the NLRP3 inflammasome to recruit caspase-8 as an alternative effector protease, thereby promoting its cleavage and activation following TNFα/cycloheximide treatment. Although previous studies have demonstrated that caspase-8 could proteolytically process pro-IL-1β [368, 369], TNFα/cycloheximide treatment of TECs did not induce IL-1β activation [364], suggesting that caspase-8 activation does not necessarily result in IL-1β processing. Interestingly, LPS priming coupled with canonical NLRP3 stimuli, including nigericin, ATP, and MSU, failed to activate caspase-8 in epithelial cells [364]. These findings contrast with observations in macrophages, where canonical NLRP3 stimuli induced caspase-8 activation [331], highlighting an additional cell type-specific difference in inflammasome signaling. Together, these findings underscore the bidirectional relationship between apoptosis-associated signaling and inflammasome activation, in which the NLRP3 inflammasome not only responds to cell death signals but also modulates downstream caspase activation in a cell type-dependent manner.

#### The NLRP3 inflammasome and PANoptosis

As discussed above, many studies have demonstrated that the NLRP3 inflammasome can engage multiple cell death pathways in response to diverse stimuli. It was recently proposed that during NLRP3 inflammasome activation, a single signaling complex, termed the PANoptosome, can be assembled to coordinate the induction of all three major forms of programmed cell death, namely, pyroptosis, apoptosis, and necroptosis, in a process collectively termed PANoptosis [370]. PANoptosis was first described in the context of influenza virus infection, where the innate immune sensor ZBP1, which recognizes cytosolic Z-DNA and Z-RNA [371, 372], was shown to activate the NLRP3 inflammasome and drive the assembly of the PANoptosome [370, 372]. Other stimuli have also been reported to induce PANoptosis downstream of NLRP3 activation. For example, prolonged stimulation of BMDMs with LPS and ATP (6 h) induced the cleavage of caspase-1, caspase-3, caspase-7, and caspase-8, accompanied by IL-1β secretion and cell death in an NLRP3- and ASC-dependent manner [373]. Consistent with the proposed PANoptosome model, a complex containing NLRP3, ASC, caspase-8, and RIPK3 was detected [373]. The PANoptosis model suggests that innate immune signaling pathways can coordinate multiple cell death programs through a shared molecular platform and may fundamentally change our understanding of programmed cell death by shifting the focus from individual pathways to their coordinated regulation. Despite these observations, clear criteria for defining PANoptosis and PANoptosome complexes are still needed to distinguish PANoptosis from the simultaneous activation of multiple cell death pathways with distinct kinetics and regulatory mechanisms [293, 374, 375]. Potential criteria may include the following: first, the signaling components of multiple cell death pathways should coexist within a single protein complex, verified by coimmunoprecipitation and cell biological imaging; second, the constituent cell death pathways should be synergistic and interdependent rather than activated in parallel, which can be assessed by genetic or pharmacologically inhibiting individual pathways and examining the effects on the others; and third, the PANoptosome can be structurally defined at the macromolecular level by cryo-electron tomography (cryo-ET) or cryo-electron microscopy (cryo-EM). Complementing these criteria, time-course analyses examining the sequential recruitment of PANoptosome components would help clarify how distinct cell death programs are coordinated and whether their coactivation reflects true interdependence or coincidental cooccurrence downstream of NLRP3 inflammasome activation.

#### GSDMD deficiency shifts NLRP3 signaling outcomes to apoptosis

GSDMD was identified as the executioner of pyroptosis and a key mediator of IL-1β secretion downstream of inflammasome activation [16–18]. In one of the early studies establishing this role, LPS plus nigericin treatment induced pyroptosis in wild-type cells but apoptosis in GSDMD-deficient cells [17]. This apoptotic response was dependent on NLRP3 and ASC [17]. Consistent with these findings, LPS plus nigericin treatment induced the activation of caspase-8, caspase-3, and caspase-7 in GSDMD-deficient macrophages but not in wild-type or NLRP3-deficient macrophages [17]. Similarly, activation of the AIM2 or NAIP–NLRC4 inflammasome in GSDMD-deficient thioglycolate-elicited peritoneal macrophages (TEPMs) resulted in delayed but significant cell death with an apoptotic morphology, in contrast to the complete absence of cell death observed in ASC- or caspase-1-deficient TEPMs [376]. These observations demonstrated that inflammasome signaling has the potential to engage both pyroptotic and apoptotic cell death pathways and that GSDMD deficiency shifts cell fate from pyroptosis to apoptosis (Fig. 4c), analogous to how caspase-8 deficiency redirects extrinsic apoptotic signaling toward necroptosis.

To elucidate the underlying molecular mechanisms, RAW 264.7 cells, a murine macrophage-like cell line lacking endogenous ASC expression, were challenged with inflammasome stimuli. Caspase-3 cleavage was detected in *Gsdmd*^−/−^ but not in wild-type or *Casp1*^−/−^*Gsdmd*^−/−^ RAW264.7 cells [376]. These findings indicated that in the absence of GSDMD, caspase-3 activation occurred in a caspase-1-dependent but ASC-independent manner. Furthermore, caspase-3 was required for caspase-1-driven apoptosis in the absence of GSDMD [376]. These results suggest that although caspase-1 is classically associated with pyroptosis, it can also promote apoptotic cell death, with GSDMD serving as a key determinant of whether inflammasome signaling leads to pyroptosis or apoptosis. This interpretation is consistent with the findings of other studies demonstrating that caspase-1 could also induce apoptosis [377–381]. The control of the cell death switch between pyroptosis and apoptosis by GSDMD was mediated through its pore-forming activity, as the expression of the pore-defective mutant GSDMD I125N also shifted the outcome toward apoptotic morphology [376]. In the absence of GSDMD activity, cleavage of multiple caspases, including caspase-3, caspase-7, caspase-8, and caspase-9, was observed. However, only genetic depletion of caspase-9 markedly reduced apoptosis and caspase-3/7 activity in this context [376]. Therefore, in the absence of GSDMD, caspase-1 was released to drive caspase-9 activation, which in turn activates caspase-3 and caspase-7 to trigger apoptosis.

The switch to apoptosis is important in the context of cells and tissue with low or no expression of endogenous GSDMD. Bone marrow–derived mast cells expressed significantly lower levels of GSDMD than BMDMs did [376]. Accordingly, nigericin-induced NLRP3 inflammasome activation in LPS-primed mast cells resulted in caspase-1-dependent apoptosis [376]. In vivo, the spinal cord expressed high levels of caspase-1 and Bid but extremely low levels of GSDMD [376], and it has been proposed that caspase-1-mediated apoptotic signaling may contribute to disease pathogenesis under conditions such as familial amyotrophic lateral sclerosis and ischemia-induced brain injury, both of which have been shown to require caspase-1 activity [378, 382–385].

In contrast to the studies discussed above, a recent study proposed that the cell death observed upon GSDMD loss may represent a secondary form of pyroptosis rather than apoptosis on the basis of the absence of characteristic apoptotic membrane blebbing despite the activation of apoptotic caspases [386]. This interpretation is supported by the detection of cleavage of GSDME, another pore-forming executioner related to GSDMD [376], and previous reports showing that caspase-3-mediated cleavage of GSDME could trigger secondary necrosis or pyroptosis [387, 388]. Further biochemical, cellular, and structural studies, such as genetic or pharmacological inhibition of GSDME and examination of other apoptotic hallmark features, including apoptotic body formation, are needed to better define the nature of this form of cell death.

#### Other perturbations shift NLRP3 signaling toward apoptosis

In addition to GSDMD deficiency, caspase-1 deficiency can also shift inflammasome-driven cell death toward apoptosis under certain conditions. The AIM2 inflammasome was reported to recruit caspase-8 in caspase-1-deficient cells [366]. Similarly, LPS-primed and nigericin-stimulated *Casp1*^−/−^ BMDMs, but not wild-type BMDMs, exhibited robust cleavage of caspase-3, caspase-8, and caspase-9 after these proteins were recruited to the NLRP3-ASC speck in *Casp1*^−/−^ BMDMs [389]. Unlike caspase-1, caspase-8 lacks a CARD and therefore did not directly interact with the CARD of ASC. Instead, caspase-8 interacted with the PYD of ASC [365, 390], which may explain why caspase-8 and caspase-1 did not compete for identical binding interfaces within the inflammasome complex [386]. In addition to promoting apoptosis, recruited caspase-8 was also capable of processing IL-1β, although with lower efficiency than caspase-1 does [391]. These findings indicate that, similar to GSDMD deficiency, caspase-1 deficiency can redirect inflammasome signaling toward apoptosis through distinct molecular mechanisms.

Other perturbations of inflammasome signaling can also divert cellular outcomes toward apoptosis. In BMDMs, prolonged nigericin treatment and low-dose DNA transfection activated NLRP3 and AIM2 inflammasomes, respectively, and both triggered apoptosis in a manner dependent on ASC but not caspase-1[365]. This activity did not require the intrinsic apoptosis pathway, as BCL-2 overexpression, which blocked mitochondrial outer membrane permeabilization, did not affect caspase-3 cleavage induced by DNA transfection [365]. Mechanistically, caspase-8, but not caspase-9, was required for caspase-3 activation with low-dose DNA transfection, although this did not lead to substantial IL-1β processing [365]. In another study [389], a delayed NLRP3 activation system was established by priming BMDMs with LPS for 4 h followed by a 12-h resting period prior to nigericin stimulation. Compared with immediately stimulated cells, delayed-stimulation cells exhibited reduced caspase-1 activation despite intact NLRP3 and pro-IL-1β induction [389]. Instead, these cells displayed caspase-8 cleavage but lacked detectable caspase-3 or caspase-9 activation [389], suggesting that delayed inflammasome activation may bias signaling toward apoptotic caspase engagement. Additional controls, including LPS priming followed by rest without secondary stimulation, can help determine whether caspase-8 activation arises from priming and resting alone.

#### Intrinsic apoptosis in NLRP3 inflammasome activation

Mitochondria play a central role in intrinsic apoptosis, as cytochrome c release is critical for caspase activation and downstream apoptotic execution [292]. The role of intrinsic (mitochondrial) apoptosis as a common essential signal in NLRP3 inflammasome activation has been debated. Early studies proposed that mitochondrial apoptosis was required for the release of oxidized mitochondrial DNA, which engaged NLRP3 and promotes its activation by canonical stimuli such as nigericin and ATP [43]. However, a later study revealed that LPS-primed *Bak*^−/−^*Bax*^−/−^ BMDMs, which lack key executioners of mitochondrial outer membrane permeabilization, did not exhibit any defects in caspase-1 activation or IL-1β processing after stimulation with diverse NLRP3 activators, including alum, imiquimod, nigericin, and ATP [48]. Similarly, the loss of BOK, another proapoptotic BCL-2 family member, or caspase-9, the initiator caspase of intrinsic apoptosis, also had no effect on NLRP3 inflammasome activation [48]. Moreover, macrophages from BCL-2–overexpressing transgenic mice, in which mitochondrial apoptosis was inhibited, similarly showed no defect in inflammasome activation [48]. Together, these findings indicate that the activation of intrinsic apoptosis is not generally required for NLRP3 inflammasome activation, although it may contribute to NLRP3 activation under specific stimuli.

Interestingly, a recent study [50] reported that inhibition of intrinsic apoptosis may, in turn, contribute to NLRP3 inflammasome activation. In both human and mouse macrophages, stimulation with NLRP3 stimuli, including nigericin, ATP, imiquimod, and CL097, blocked apoptosis induced by intrinsic apoptosis activators such as raptinal, BH3 mimetics, or staurosporine [50]. In contrast, these NLRP3 stimuli did not inhibit extrinsic apoptosis [50]. The suppression of intrinsic apoptosis was independent of NLRP3 and ASC [50], indicating that it occurred upstream of inflammasome assembly. These stimuli may interfere with mitochondrial ATP production, potentially through alterations in mitochondrial crista morphology [50]. However, inhibition of intrinsic apoptosis or mitochondrial ATP production alone was insufficient to activate the NLRP3 inflammasome [50]. Instead, inflammasome activation required a second signal, such as resiquimod, which promoted the dispersion of the TGN into vesicles and enabled the recruitment of NLRP3 [50]. These findings suggest that alternations in mitochondrial ATP production associated with the inhibition of intrinsic apoptosis may function as an additional parallel signal required for full NLRP3 activation, complementing previously described inputs such as K⁺ efflux and TGN remodeling.

In summary, the crosstalk between the NLRP3 inflammasome and other innate immune signaling and cell death pathways is complex and context-dependent; however, several common themes have emerged. First, because NLRP3 activation follows a two-step process, other innate immune and cell death pathways can intersect with NLRP3 signaling at either step or both steps (e.g., NLRP1 and NLRP12). Second, NLRP3 responds to common cellular danger signals, including K⁺ efflux and lysosomal rupture, indicating that other signaling pathways can indirectly engage NLRP3 by triggering these events. For example, cGAS–STING activation in human myeloid cells induced lysosomal damage and K⁺ efflux, thereby engaging the NLRP3 inflammasome as a downstream effector. Third, because NLRP3 shares domain architecture with other inflammasome sensors and because all inflammasomes converge on common downstream components, multiple sensors can functionally synergize. Indeed, inflammasome receptors such as NAIP–NLRC4 and NLRP12 can incorporate NLRP3 into a shared complex, amplifying or sustaining activation and revealing a broader principle by which inflammasome pathways cooperate rather than operate in isolation. Finally, NLRP3 inflammasome activation does not simply culminate in a single outcome but instead licenses a range of downstream responses, including the engagement of the apoptotic cell death pathway, illustrating how NLRP3 acts as a hub that coordinates multiple inflammatory and cell death programs.

## Conclusion and perspectives

The NLRP3 inflammasome is now recognized as a central signaling hub that integrates inputs from multiple innate immune and cell death pathways rather than functioning as an isolated sensor. Despite substantial progress, several important questions remain unanswered.

First, how pathogens engage multiple innate immune pathways in a coordinated manner remains poorly understood. Individual pathogens often generate diverse PAMPs and induce distinct forms of cellular stress, thereby activating several sensing pathways simultaneously. The kinetics, magnitude, and subcellular localization of these signals likely determine whether pathways cooperate, antagonize one another, or engage distinct downstream programs. Furthermore, these interactions are likely highly cell type specific, as different immune and nonimmune cell populations possess distinct signaling thresholds and effector repertoires. Future studies using single-cell and spatiotemporal approaches will be essential for determining how signaling dynamics shape inflammasome outcomes during infection.

Second, most studies on NLRP3 crosstalk have been performed in the context of infectious diseases, whereas the relevance of these interactions in sterile inflammation remains largely unexplored. This knowledge gap is particularly important because aberrant NLRP3 activation has been implicated in neurodegenerative disorders, metabolic diseases, cardiovascular diseases, and cancer [6, 7, 392]. Whether pathways such as cGAS–STING, RIG-I/MDA5–MAVS signaling, or regulated cell death programs contribute to NLRP3 activation and pathology in these settings remains an open question with significant therapeutic implications. Most current therapeutic strategies target individual pathways, yet the highly interconnected nature of NLRP3 signaling and other innate immune and inflammatory signaling pathways suggests that combined or context-dependent approaches may be required to effectively treat inflammasome-driven diseases.
